# ECB unconventional monetary policy and SME access to finance

**DOI:** 10.1007/s11187-023-00730-0

**Published:** 2023-02-18

**Authors:** Marie Finnegan, Supriya Kapoor

**Affiliations:** 1 Atlantic Technological University, Galway, Ireland; 2 Trinity College Dublin, Dublin, Ireland; 3 Technological University Dublin, Dublin, Ireland

**Keywords:** Unconventional monetary policy, SMEs, Credit access, Bank lending, Micro firms, E52, E58, D22, L26

## Abstract

Small- and medium-sized enterprises (SMEs) account for two-thirds of employment in the euro area which makes them a priority for the transmission of monetary policy to the real economy. SMEs in Europe experienced a credit crunch following the sovereign debt crisis. Over the period 2014–2019, the European Central Bank (ECB) engaged in unconventional monetary policy (UMP) to restore funding conditions in the euro area, to support stronger economic growth and higher inflation. We use the ECB/EC Survey on the Access to Finance of Enterprises to examine the relationship between monetary policy and SME access to finance in countries that were most affected by the crisis as follows: Greece, Ireland, Italy, Portugal and Spain. We show that the implementation of UMP increases the probability that firms with higher debt-to-assets ratio remain credit constrained in stressed countries, although this effect becomes insignificant in non-stressed countries. Our findings suggest that monetary policy is transmitted unevenly to leveraged SMEs across jurisdictions. Additionally, we find little evidence that risky firms are credit constrained during periods of UMP, when risk is measured from the firms’ own viewpoint. However, our heterogenous analysis shows that smaller and younger firms—which are also considered to be risky—remain credit constrained over this period. Policy should ensure that UMP trickles down to SMEs regardless of their size, age or location. Tweetable line: Leveraged SMEs in stressed countries are more likely to remain credit constrained even when monetary policy is expansionary. Policy must do more to support small and young firms’ access to credit to facilitate higher investment and growth.

## Abstract (plain language summary)

The global financial crisis which began in 2007 and the subsequent sovereign debt crisis which began in 2010 negatively affected many small- and medium-sized enterprises (SMEs) in accessing finance. Over the period 2014–2019, the ECB implemented various ‘unconventional monetary policy’ (UMP) tools to make it easier for companies to get loans, to boost economic growth and to bring inflation close to their 2% target. We use the ECB/EC Survey on the Access to Finance of Enterprises (SAFE) to examine the relationship between monetary policy and the ability of SMEs to access finance in countries that were most badly affected by the two crises. These are known as the ‘stressed’ countries and are Greece, Ireland, Italy, Portugal and Spain. We show that UMP increased the probability that firms in stressed countries with higher debt-to-assets ratio are credit constrained. However, this effect is insignificant in non-stressed countries. Additionally, we find little evidence that risky firms are credit constrained during periods of UMP, when risk is measured from the firms’ viewpoint. However, smaller and younger firms—which are also seen as risky—do suffer from financial constraints. There are a number of policy implications arising from this research. First, monetary policy should operate in a manner that SMEs in all euro-area countries have similar access to bank finance. Second, monetary policy should make sure that UMP assists smaller and younger firms. Finally, public policy could also intervene to support technologies to allow access to real-time transaction flows between banks and firms to allow banks to be better informed about the risk profile of firms and allocate funds to SMEs that warrant liquidity.

## Introduction

The global financial crisis (GFC) which began in 2007 and the subsequent euro area sovereign debt crisis which began in 2010 adversely affected firms’ access to finance, leading to lower economic growth and a prolonged period of low inflation (Acharya et al., 2018). Countries in the periphery^1^ suffered disproportionally from higher sovereign rates, reflecting their deteriorated sovereign creditworthiness. Empirical studies show that small- and medium-sized enterprises (SMEs) in these countries that were mostly affected by the sovereign debt crisis suffered more in terms of access to finance (Bańkowska et al., 2020; Boeckx et al., 2017; Corbisiero & Faccia, 2019). The credit crunch that followed the crisis disrupted SMEs’ business and investment activities, prolonging low economic growth and subdued inflation (Bongini et al., 2021).

In 2013–2014, euro-area inflation was still well below the European Central Bank’s (ECB’s) price stability target of below, but close to, 2% over the medium term due to the low inflation recovery following the GFC and sovereign debt crisis. Faced with zero-bound interest rates in 2014, the ECB engaged in accommodative monetary policy, commonly referred to as unconventional monetary policy (UMP), aimed at returning inflation to levels consistent with its price stability target and incentivising banks to lend to the real economy.^2^ This included tools such as forward guidance (FG), negative interest rate policy (NIRP), targeted long-term refinancing operations (TLTROs) and various asset purchase programmes (APPs), which worked together to restore funding conditions in the euro area and support higher inflation (Hartmann & Smets, 2018; Rostagno et al., 2019). A description of each of the UMP tools is highlighted in Sect. 2.

In this paper, we investigate the probability of a firm being credit constrained in the presence of UMP. The key research question we ask is whether SMEs, particularly highly leveraged and risky, in stressed countries continued to remain credit constrained even during the increase in size and scope of UMP over the period 2014–2019. We focus on SMEs as they are highly reliant on bank credit for survival and growth (Ferrando et al., 2014; Gerlach-Kristen et al., 2015), are often unable to borrow in the corporate bond market or raise capital in the stock market (Bougheas et al., 2006; Kashyap & Stein, 1994) and are likely to become credit constrained when banks adjust their loan portfolios in response to negative shocks to their balance sheets (Duygan-Bump et al., 2015; Khwaja & Mian, 2008).

Further, SMEs accounted for 99.8% of firms in Europe, 53% of the total value added and 65% of total employment in the EU in 2021 and are central in supporting economic growth, innovation, job creation and social integration (EC, 2022). Specifically, we focus on SMEs in the five euro-area countries: Greece, Ireland, Italy, Portugal and Spain (hereafter denoted as ‘stressed countries’) that experienced deteriorated credit access following the GFC and the sovereign debt crisis. The rationale for choosing these countries is threefold. First, stressed countries experienced a substantial deterioration in their sovereign creditworthiness, while the rest of the countries in the euro area did not. With banks holding large quantities of debt securities issued domestically, investors lost faith in the banking sectors of stressed countries, pushing banks’ funding costs up (Albertazzi et al., 2014; Bańkowska et al., 2020). Second, banks in stressed countries suffered from relatively higher non-performing loans and lower capital ratios, which further restrained lending to SMEs as banks sought to repair their balance sheets in the wake of tighter macro-prudential policy triggered by the GFC (Altavilla et al., 2019). Third, SMEs in stressed countries had accumulated significant debt prior to the GFC, mainly related to real estate in countries such as Spain and Ireland, which impacted their access to credit (Cussen & O'Leary, 2013; Fernández de Guevara et al., 2021). Therefore, the impact of UMP on SMEs’ credit constraints in stressed countries remains an important empirical question. However, for a comprehensive analysis on SMEs, we also compare our results for stressed countries to non-stressed countries.

We add to the literature by assessing the impact of UMP over the period 2014 to 2019 on SMEs in stressed and non-stressed countries. Particularly, we examine two related hypotheses to determine if UMP trickled down to SMEs. First, we assess if the implementation of UMP reduces the probability that firms with increased debt-to-assets ratio (high leveraged) are credit constrained. Second, we investigate if the implementation of UMP reduces the probability of firms being credit constrained using firm-level measures of risk. To test both hypotheses, we construct a measure of monetary policy at the country level following Peydró et al. (2021). Specifically, we take the logarithm of total assets from the individual country’s central bank balance sheets minus the autonomous factors such as banknotes in circulation and government balances at the central bank.

The literature on the transmission of monetary policy to the real economy generally focuses on the impact of monetary policy on the asset side of bank balance sheets and how balance sheet health alters this impact (Bernanke & Blinder, 1988; Kapoor & Peia, 2021). However, given that firms in Europe are relatively bank dependent, the ECB’s inflation target is also dependent on their firm-level decision process (Anastasiou & Giannoulakis, 2022). Therefore, we contribute to the growing literature on the impact of UMP on firm financing decisions using a micro firm-based survey that was designed to measure loan demand. Our paper also relates to a number of different literatures. First is the impact of leverage on firms’ access to finance (Corbisiero & Faccia, 2019; Ferrando & Mulier, 2015; Kaya & Masetti, 2018). On the one hand, high leveraged firms might feel unconstrained as they hold a lot of debt on their balance sheets, but on the other hand, this might make it difficult or costly for firms to find new debt (Durante et al., 2020).

Second, this paper considers the heterogenous effect of monetary policy on firms with different leverage and risk profiles (Caglio, 2021; Bianco, 2021). Much of the empirical literature which assesses heterogeneity in firm leverage and access to finance due to monetary policy concern publicly listed US firms and mainly study periods of conventional monetary policy (Jeenas, 2019; Ottonello and Winberry, 2020; Aktar et al., 2021). Other research does focus on SMEs’ access to finance in the euro area but does not consider the interaction of leverage and UMP on firms’ credit constraints (Boeckx et al., 2014; Burriel and Galesi, 2018; Corbisiero & Faccia, 2019; Bańkowska et al., 2020). This is despite leverage being cited as a reason for relatively poor access to finance for firms in stressed countries during the two crises (Cussen & O'Leary, 2013; Fernández de Guevara et al., 2021). This research adds to the literature by considering the sensitivity of leveraged firms to UMP in influencing credit constraints in SMEs in the euro area, during the period 2014 to 2019 when UMP was increasing in size and scope.

Third, most of the monetary policy transmission literature that considers risk focuses on objective measures of risk and if banks reallocate their asset portfolio towards these risky assets. Objective measures include firm size and age, which increase screening costs for banks (Berger & Udell, 1998; Bernanke et al., 1996; De Jonghe et al., 2020; Calabrese et al., 2021), banks’ internal ratings on loans to businesses (Dell'ariccia et al., 2017; Jimenez et al., 2014), macroeconomic variables to capture the economic outlook as worsening of economic outlook leads to deterioration in borrowers’ creditworthiness and increases credit risk (Burlon et al., 2019; Maddaloni & Peydro, 2011), the firm Z-scores (Jiménez et al., 2018; Peydró et al., 2021), bank write-offs to total loans (De Jonghe et al., 2020) or loan yield (Peydró et al., 2021). We build on this literature by using a future predictor of risk, profit decreased in the previous 6 months as well as a selection of subjective measures of risk such as the firms’ own view if their credit history, own economic outlook or own capital has deteriorated in the previous 6 months and, finally, an activity-based measure of risk, i.e. innovative activity, given that such activity is more uncertain and, therefore, riskier. Calabrese et al. (2021) use firm subjective measures of risk such as the firms’ view of their own capital and credit history but consider them in the context of financial fragmentation. To the best of our knowledge, this paper provides the first piece of evidence on the use of firm-based measures of risk to identify SME access to funding in an environment of UMP. The use of firm-based measures is important because if firms view themselves as risky, they may be discouraged from applying for a loan due to fear of rejection despite UMP, and this may represent an efficient market outcome. Finally, this paper considers the heterogenous effects of firms’ access to finance based on firm size and age, given the theoretical and empirical literature that shows that smaller and younger firms are more likely to be credit constrained (Berger & Udell, 1998; Bernanke et al., 1996).

We employ firm micro-level data on 11,319 SME observations from the EU/ECB’s ‘Survey on the Access to Finance of Enterprises’ (SAFE) to evaluate the relationship between UMP and SME credit access during the time period 2014–2019. The data used in the study investigates this particular time period for three reasons. First, many of the factors that had led to SMEs in stressed countries being relatively more credit constrained had receded due to expansionary monetary policy and more stringent macro-prudential policy in the aftermath of the GFC and sovereign debt crisis. Second, UMP increased in scope and scale from 2015 to 2019. For example, between September 2014 and the end of 2018, the ECB purchased over €2.5 trillion worth of securities under various APPs (Larkin et al., 2019). Finally, we focus our attention until 2019, which is the time period before the COVID-19 pandemic impacted the picture.

Our results are twofold. First, our findings show that as UMP is implemented, firms with increased debt-to-assets ratio are more likely to be credit constrained in stressed countries relative to non-stressed countries. We could argue that banks in stressed countries are more sensitive to higher leveraged firms when extending credit, even in an environment of UMP. This could be due to the legacy of the negative fallout for banks that suffered more in stressed countries from taking on excessive risk prior to the financial crisis (Blanco and Jimenez, 2018; Corbisiero & Faccia, 2019; Fernández de Guevara et al., 2021) or because banks in stressed countries may suffer from tighter regulatory constraints (Altavilla et al., 2019). Second, when firms’ risk variables from their own viewpoint are considered, we do not find any significant results implying that we cannot say whether risky firms are more or less credit constrained during UMP. This holds for both stressed and non-stressed countries. Our findings also survive a battery of sub-sample analysis and other robustness tests including various definitions of the dependent variable and the monetary policy indicator.

Our research is important from a policy perspective. In particular, the transmission of UMP to SMEs is vital, given their bank dependence and importance in terms of economic activity. Furthermore, the ECB continues to use UMP tools in times of market stress and economic downturns, and these tools will continue to remain important (Schnabel, 2021). Our findings provide some insights into the ability of UMP to provide credit to SMEs and contribute to the debate on the efficacy of these policies.

The remainder of the paper is structured as follows. Section 2 presents the institutional framework of UMP. Section 3 outlines the related literature and develops the hypotheses. We detail the dataset we use and how we build our measurement of monetary policy, provide descriptive statistics for all the variables employed in the study and present empirical methodology in Sect. 4. We analyse the effect of UMP on firms’ probability of being credit constrained in Sect. 5. Section 6 concludes.

## Institutional framework

The GFC and sovereign debt crises from 2008 to 2013 led to, inter alia, a credit crunch and substantial deflationary risks in the euro area. The ECB responded with conventional monetary policy and cut the deposit facility rate—the interest banks receive for depositing money with the central bank overnight—by a cumulative 325 bps from October 2008 falling to zero in July 2012. This zero lower bound limited the ECB’s ability to use conventional monetary policy to target price stability (Bowdler & Radia, 2012), and the ECB began to use UMP from 2010 amid persistent deflationary pressures. UMP encompasses ECB market operations focused on ensuring that market instabilities would not disrupt the transmission of the historically low policy rates to the economy (Rostagno et al., 2019). Table 1 provides a brief description of the main UMP tools employed by the ECB from 2010 to 2019, and these include the SMP, OMTs, FG, NIRP, credit operations via TLTROs and various ECB APPs. These tools work together in a ‘combined arms’ approach to ease financial constraints, address the heterogeneous transmission in bank lending across the euro area, stimulate the economy and bring back inflation to the ECB’s target (ECB, 2017; Hartmann & Smets, 2018; Rostagno et al., 2019). This section discusses each of these in turn.

**Table 1:** ECB unconventional monetary policy tools from 2010 to 2019

Policy tool	Acronym	Timeline	Definition
Securities market programme	SMP	May 2010 to September 2012	Intervention by the ECB in public and private debt securities markets to ensure depth and liquidity in malfunctioning segments of the debt securities markets and to restore an appropriate functioning of the monetary policy transmission mechanism. No injection of liquidity as fully sterilised by selling other bonds or bills (ECB, 2010)
Outright monetary transactions	OMTs	September 2012	Commitment by the ECB to buy risky sovereign debt in stressed secondary sovereign bond markets of bonds issued by euro-area countries under certain conditions. No injection of liquidity as fully sterilised by selling other bonds or bills (ECB, 2012)
Forward guidance	FG	July 2013–2019	ECB provides guidance about their expectation for future policy rates based on its assessment of the outlook for price stability to safeguard appropriate monetary policy transmission (ECB, 2014a, b)
Negative interest rate policy	NIRP	June 2014–2019	ECB introduces negative ECB deposit facility rate (DFR) to incentivise bank lending to real economy (ECB, 2014a, b)
Targeted long-term refinancing operations	TLTROs	June 2014–2019	ECB offers longer-term loans to banks at favourable costs and encourages them to lend to businesses and consumers in the euro area (ECB, 2021)
Asset purchase programmes	APPs	October 2014–2019	The ECB purchases private and public sector assets from investors such as pension funds and banks. This compresses yields across several markets and across the entire yield curve, increases asset values and makes lending more attractive for banks. Liquidity injection (ECB, 2016)

The ECB started the SMP in 2010 in a response to market instability arising from the GFC, by buying public and private debt securities in secondary markets reaching about €220 billion in February 2012. The ECB simultaneously absorbed the same amount of liquidity, a process known as sterilisation, in order to keep the monetary policy stance neutral (Eser & Schwaab, 2016). This was followed by the announcement of the OMT programme in 2012 that aimed to ensure the ECB’s monetary policy is transmitted equally to all euro-area member countries. Next, forward guidance was used from July 2013 where the ECB provided guidance about its expectation for future policy rates to anchor medium-term rates at levels more consistent with their intentions (Altavilla et al., 2021). This increases confidence around low levels of long-run real interest rates and reduces uncertainty in markets, thereby increasing credit demand and stimulating firm investment (ECB, 2014a, b). Evidence suggests that this signalling is a relatively strong channel (Bauer & Rudebusch, 2014; Eser et al., 2019) Tables 2, 3, 4, and 5.

**Table 2:** Summary statistics

Variables	Observations	Mean	SD	Minimum	Maximum
Dependent variable					
Credit constrained	11,319	0.405	0.491	0	1
Monetary policy variables					
National central bank assets less autonomous factors	11,319	0.194	0.105	0.00573	0.335
National central bank government securities	11,319	0.174	0.145	0.0122	0.470
National central bank government securities + MFI securities + MFI lending	11,319	0.331	0.229	0.0262	0.758
Firm balance sheet variable					
Debt-to-assets ratio decreased	11,116	0.307	0.461	0	1
Firm risk variables					
Profit decreased	11,180	0.408	0.491	0	1
Own outlook deteriorated	11,181	0.315	0.465	0	1
Own capital deteriorated	11,211	0.148	0.355	0	1
Credit history deteriorated	11,252	0.150	0.357	0	1
Innovation	11,319	0.332	0.471	0	1
Bank characteristic variables					
Non-performing loans (%)	11,319	16.01	11.26	3.390	45.81
Regulatory tier 1 capital as a share of risk-weighted assets (%)	11,319	14.00	2.994	10.59	25.21
Macroeconomic variables					
Inflation (%)	11,319	0.433	0.880	− 2.017	2.050
Unemployment (%)	11,319	16.49	5.639	5	26.60
Firm characteristic variables					
Micro	11,319	0.400	0.490	0	1
Small	11,319	0.330	0.470	0	1
Medium	11,319	0.270	0.444	0	1
Turnover up to 2 mn	11,176	0.536	0.499	0	1
Turnover between 2 and 10 mn	11,176	0.256	0.437	0	1
Turnover between 10 and 50 mn	11,176	0.177	0.382	0	1
More than 10 years	11,312	0.865	0.342	0	1
Between 5 and 10 years	11,312	0.0953	0.294	0	1
Between 2 and 5 years	11,312	0.0321	0.176	0	1
Less than 2 years	11,312	0.00796	0.0888	0	1
Stand-alone firm	11,319	0.941	0.236	0	1
Individual or family owned	11,309	0.869	0.338	0	1
Industry	11,319	0.576	0.494	0	1
Trade	11,319	0.331	0.471	0	1
Construction	11,319	0.0926	0.290	0	1

Summary statistics recorded throughout the sample. Variable definitions are provided in Appendix 2

*mn* million

**Table 3:** Probability of being credit constrained on monetary policy and debt-to-assets ratio, stressed versus non-stressed countries

Credit-constrained variables	Stressed				Non-stressed			
	1	2	3	4	5	6	7	8
MP_*t* − 2_	− 0.00886 (0.0240)	− 0.0181 (0.0263)	− 0.0327 (0.0299)	− 0.0309 (0.0286)	0.00977 (0.0246)	0.00658 (0.0249)	0.0558* (0.0313)	0.0456 (0.0306)
Debt-to-assets ratio increased	− 0.546*** (0.136)	− 0.551*** (0.136)	− 0.551*** (0.136)	− 0.460*** (0.130)	0.149 (0.107)	0.146 (0.107)	0.148 (0.107)	0.115 (0.104)
MP_*t* − 2_ × debt-to-assets ratio increased	0.0491*** (0.0114)	0.0495*** (0.0114)	0.0495*** (0.0114)	0.0417*** (0.0109)	− 0.00916 (0.00888)	− 0.00894 (0.00885)	− 0.00907 (0.00886)	− 0.00684 (0.00863)
Bank characteristic variables								
Non-performing loans_*t* − 2_		− 0.00234 (0.00188)	− 0.00281 (0.00211)	− 0.00256 (0.00200)		0.00616 (0.00894)	0.00139 (0.00923)	0.00433 (0.00897)
Tier 1 capital ratio_*t* − 2_		− 0.00238 (0.00599)	− 0.00506 (0.00652)	− 0.00418 (0.00617)		− 0.00164 (0.00378)	− 0.00179 (0.00382)	− 0.00243 (0.00372)
Macroeconomic variables								
Unemployment_*t* − 2_			0.00148 (0.00170)	0.00140 (0.00164)			0.0421** (0.0180)	0.0386** (0.0174)
Inflation_*t* − 2_			0.0135 (0.0143)	0.0113 (0.0138)			0.0218 (0.0157)	0.0172 (0.0152)
Firm characteristic variables								
Micro				0.0932*** (0.0180)				0.0866*** (0.0169)
Small				0.0114 (0.0153)				0.0295** (0.0135)
Trade				− 0.0366 (0.0289)				− 0.0473 (0.0446)
Industry				− 0.0832*** (0.0285)				− 0.00561 (0.0438)
Less than 2 years				0.0867* (0.0519)				− 0.00157 (0.0390)
Between 2 and 5 years				0.126*** (0.0257)				0.0642*** (0.0233)
Between 5 and 10 years				0.0162 (0.0159)				0.0424*** (0.0144)
Turnover up to 2 mn				0.303*** (0.0377)				0.213*** (0.0269)
Turnover 2–10 mn				0.180*** (0.0364)				0.131*** (0.0252)
Turnover 10–50 mn				0.0685* (0.0365)				0.0419* (0.0246)
Individual or family owned				− 0.00874 (0.0153)				− 0.00840 (0.0127)
Stand-alone firm				− 0.0966*** (0.0215)				− 0.0190 (0.0146)
Observations	8777	8777	8777	8668	8168	8168	8168	8024
Country × sector FE	Yes	Yes	Yes	Yes	Yes	Yes	Yes	Yes
Time FE	Yes	Yes	Yes	Yes	Yes	Yes	Yes	Yes
Bank controls	No	Yes	Yes	Yes	No	Yes	Yes	Yes
Macro controls	No	No	Yes	Yes	No	No	Yes	Yes
Other firm controls	No	No	No	Yes	No	No	No	Yes

The probability of being credit constrained is the dependent variable for stressed countries in columns 1–4 and for non-stressed countries in columns 5–8. MP_*t* − 2_ is 1-year lag (equivalent to two survey waves). Debt-to-assets ratio increased is a categorical variable which is equal to 1 if the firm’s debt-to-assets ratio increased, and 0 if it remained the same or decreased in the previous 6 months. Bank controls (non-performing loans and tier 1 capital ratio) and macro controls (inflation and unemployment) are lagged by 1 year (equivalent to two survey waves). Robust standard errors are in the parentheses.

*mn* million.

*Significance at 10%

**Significance at 5%

***Significance at 1%

**Table 4:** Probability that the implementation of UMP leads to risky firms being less credit constrained in stressed countries

Credit-constrained variables	1	2	3	4	5	6	7	8	9	10
MP_*t* − 2_	− 0.00733 (0.0294)	− 0.00823 (0.0282)	− 0.00871 (0.0284)	− 0.00923 (0.0274)	− 0.00458 (0.0289)	− 0.00648 (0.0278)	− 0.0179 (0.0287)	− 0.0171 (0.0277)	− 0.0159 (0.0297)	− 0.0169 (0.0283)
Profit decreased	0.0377 (0.124)	0.0657 (0.120)								
MP_*t* − 2_ × profit decreased	0.0110 (0.0104)	0.00521 (0.0101)								
Credit history deteriorated			0.105 (0.209)	0.0990 (0.205)						
MP_*t* − 2_ × credit history			0.0151 (0.0174)	0.0113 (0.0170)						
Own outlook deteriorated					0.174 (0.140)	0.114 (0.135)				
MP_*t* − 2_ × own outlook					0.00654 (0.0117)	0.00836 (0.0113)				
Own capital deteriorated							− 0.0434 (0.175)	− 0.0611 (0.169)		
MP_*t* − 2_ × own capital							0.0274* (0.0148)	0.0234 (0.0143)		
Innovation									− 0.119 (0.127)	− 0.0960 (0.122)
MP_*t* − 2_ × innovation									0.0111 (0.0107)	0.00955 (0.0103)
Bank characteristic variables										
Non-performing loans_*t* − 2_	− 0.000188 (0.00205)	− 0.000596 (0.00197)	− 0.00131 (0.00203)	− 0.00132 (0.00194)	0.00530*** (0.00205)	0.00421** (0.00197)	− 0.00149 (0.00204)	− 0.00173 (0.00196)	− 0.00247 (0.00208)	− 0.00228 (0.00198)
Regulatory tier capital ratio_*t* − 2_	− 0.00247 (0.00635)	− 0.00194 (0.00605)	− 0.00183 (0.00626)	− 0.00147 (0.00598)	0.00356 (0.00624)	0.00295 (0.00598)	− 0.00342 (0.00630)	− 0.00290 (0.00603)	− 0.00192 (0.00645)	− 0.00178 (0.00610)
Macroeconomic variables										
Unemployment_*t* − 2_	0.00147 (0.00167)	0.00145 (0.00162)	0.00184 (0.00164)	0.00174 (0.00159)	0.00169 (0.00164)	0.00169 (0.00159)	0.00169 (0.00166)	0.00147 (0.00161)	0.00135 (0.00169)	0.00130 (0.00163)
Inflation_*t* − 2_	0.00583 (0.0140)	0.00527 (0.0135)	0.0103 (0.0138)	0.00832 (0.0133)	− 0.00453 (0.0137)	− 0.00362 (0.0133)	0.0146 (0.0138)	0.0118 (0.0133)	0.0123 (0.0142)	0.00961 (0.0137)
Firm characteristic variables										
Micro		0.0839*** (0.0178)		0.0777*** (0.0175)		0.0740*** (0.0175)		0.0790*** (0.0176)		0.0916*** (0.0179)
Small		0.00956 (0.0151)		0.00351 (0.0148)		0.00190 (0.0148)		0.00822 (0.0149)		0.00871 (0.0152)
Trade		− 0.0393 (0.0282)		− 0.0330 (0.0283)		− 0.0413 (0.0280)		− 0.0305 (0.0280)		− 0.0346 (0.0285)
Industry		− 0.0914*** (0.0278)		− 0.0735*** (0.0278)		− 0.0925*** (0.0276)		− 0.0805*** (0.0276)		− 0.0855*** (0.0282)
Less than 2 years		0.107** (0.0506)		0.0807 (0.0507)		0.0894* (0.0516)		0.0918* (0.0505)		0.0868* (0.0525)
Between 2 and 5 years		0.123*** (0.0252)		0.129*** (0.0255)		0.111*** (0.0249)		0.126*** (0.0254)		0.124*** (0.0256)
Between 5 and 10 years		0.0256 (0.0156)		0.0231 (0.0156)		0.0167 (0.0155)		0.0216 (0.0158)		0.0160 (0.0158)
Turnover up to 2 mn		0.271*** (0.0370)		0.272*** (0.0365)		0.250*** (0.0367)		0.278*** (0.0366)		0.295*** (0.0372)
Turnover between 2 and 10 mn		0.155*** (0.0357)		0.157*** (0.0350)		0.143*** (0.0353)		0.168*** (0.0351)		0.171*** (0.0358)
Turnover between 10 and 50 mn		0.0572 (0.0358)		0.0556 (0.0351)		0.0389 (0.0354)		0.0634* (0.0352)		0.0589 (0.0359)
Stand-alone firm		− 0.0942*** (0.0212)		− 0.0912*** (0.0206)		− 0.0974*** (0.0211)		− 0.0905*** (0.0210)		− 0.0964*** (0.0214)
Observations	8836	8726	8896	8779	8826	8707	8849	8734	8943	8820
Country × sector FE	Yes	Yes	Yes	Yes	Yes	Yes	Yes	Yes	Yes	Yes
Time FE	Yes	Yes	Yes	Yes	Yes	Yes	Yes	Yes	Yes	Yes
Bank controls	Yes	Yes	Yes	Yes	Yes	Yes	Yes	Yes	Yes	Yes
Macro controls	Yes	Yes	Yes	Yes	Yes	Yes	Yes	Yes	Yes	Yes
Other firm controls	No	Yes	No	Yes	No	Yes	No	Yes	No	Yes

The dependent variable in columns 1–10 is the probability of being credit constrained for firms in stressed countries. MP_*t* − 2_ is the 1-year lag (equivalent to two survey waves) of the logarithm of the assets of individual central bank balance sheets—minus the autonomous factors—for stressed countries. Profit decreased, credit history deteriorated, own outlook deteriorated and own capital deteriorated are all categorical variables which proxy firm risk from the firm’s viewpoint. Innovation is a categorical variable which proxies if the firm innovated in the previous 6 months, and is a measure of firm risk. Country sector fixed effects, time sector fixed effects, bank controls and macro controls (both lagged by 1 year, equivalent to two survey waves) are included in all specifications. Firm controls are added in columns 2, 4, 6, 8 and 10. Robust standard errors are in parentheses.

*mn* million.

*Significance at 10%

**Significance at 5%

***Significance at 1%

**Table 5:** Probability that the implementation of UMP leads to risky firms being less credit constrained in non-stressed countries

Credit-constrained variables	1	2	3	4	5	6	7	8	9	10
MP_*t* − 2_	0.0466 (0.0311)	0.0406 (0.0304)	0.0444 (0.0301)	0.0364 (0.0295)	0.0593* (0.0313)	0.0525* (0.0304)	0.0535* (0.0298)	0.0482* (0.0293)	0.0453 (0.0315)	0.0387 (0.0307)
Profit decreased	0.0448 (0.100)	0.0469 (0.0979)								
MP_*t* − 2_ × profit decreased	0.00596 (0.00825)	0.00480 (0.00804)								
Credit history deteriorated			0.276** (0.132)	0.231* (0.127)						
MP_*t* − 2_ × credit history			− 0.00552 (0.0108)	− 0.00380 (0.0105)						
Own outlook deteriorated					0.227** (0.105)	0.237** (0.102)				
MP_*t* − 2_ × own outlook					− 0.00145 (0.00861)	− 0.00423 (0.00841)				
Own capital deteriorated							0.168 (0.119)	0.196* (0.116)		
MP_*t* − 2_ × own capital							0.00547 (0.00978)	0.000221 (0.00954)		
Innovation									− 0.0292 (0.109)	− 0.00977 (0.107)
MP_*t* − 2_ × innovation									0.00588 (0.00886)	0.00496 (0.00865)
Bank characteristic variables										
Non-performing loans_*t* − 2_	− 0.000607 (0.00912)	0.00168 (0.00886)	− 0.00260 (0.00898)	0.000233 (0.00877)	− 0.0151* (0.00904)	− 0.0108 (0.00879)	− 0.00279 (0.00888)	− 0.000137 (0.00872)	0.00195 (0.00919)	0.00484 (0.00892)
Regulatory tier capital ratio_*t* − 2_	− 0.000788 (0.00377)	− 0.00182 (0.00367)	− 0.00128 (0.00370)	− 0.00181 (0.00362)	0.00163 (0.00369)	0.000409 (0.00360)	0.000505 (0.00366)	− 0.000255 (0.00360)	− 0.00122 (0.00382)	− 0.00209 (0.00372)
Macroeconomic variables										
Unemployment_*t* − 2_	0.0419** (0.0177)	0.0403** (0.0171)	0.0421** (0.0173)	0.0388** (0.0168)	0.0664*** (0.0178)	0.0612*** (0.0172)	0.0495*** (0.0170)	0.0456*** (0.0165)	0.0397** (0.0180)	0.0367** (0.0174)
Inflation_*t* − 2_	0.0221 (0.0155)	0.0179 (0.0151)	0.0273* (0.0153)	0.0224 (0.0149)	0.0200 (0.0153)	0.0159 (0.0149)	0.0217 (0.0151)	0.0174 (0.0147)	0.0206 (0.0155)	0.0162 (0.0151)
Firm characteristic variables										
Micro		0.0806*** (0.0167)		0.0866*** (0.0164)		0.0660*** (0.0164)		0.0780*** (0.0164)		0.0923*** (0.0166)
Small		0.0256* (0.0134)		0.0303** (0.0131)		0.0225* (0.0131)		0.0306** (0.0132)		0.0310** (0.0134)
Industry		− 0.0106 (0.0437)		0.00681 (0.0436)		− 0.00465 (0.0427)		− 0.000237 (0.0427)		− 0.00401 (0.0433)
Less than 2 years		0.0145 (0.0393)		− 0.0106 (0.0371)		0.00456 (0.0371)		0.00228 (0.0367)		− 0.0115 (0.0378)
Between 2 and 5 years		0.0691*** (0.0230)		0.0560** (0.0227)		0.0646*** (0.0226)		0.0576** (0.0224)		0.0651*** (0.0231)
Between 5 and 10 years		0.0440*** (0.0141)		0.0434*** (0.0139)		0.0444*** (0.0140)		0.0430*** (0.0138)		0.0400*** (0.0141)
Turnover up to 2 mn		0.205*** (0.0269)		0.192*** (0.0265)		0.204*** (0.0267)		0.181*** (0.0264)		0.210*** (0.0267)
Turnover between 2 and 10 mn		0.124*** (0.0252)		0.115*** (0.0249)		0.124*** (0.0250)		0.110*** (0.0247)		0.127*** (0.0251)
Turnover between 10 and 50 mn		0.0325 (0.0247)		0.0336 (0.0244)		0.0396 (0.0245)		0.0313 (0.0241)		0.0405* (0.0246)
Stand-alone firm		− 0.0246* (0.0143)		− 0.0226 (0.0141)		− 0.0244* (0.0142)		− 0.0223 (0.0140)		− 0.0215 (0.0143)
Observations	8176	8032	8319	8159	8217	8061	8320	8164	8389	8221
Country × sector FE	Yes	Yes	Yes	Yes	Yes	Yes	Yes	Yes	Yes	Yes
Time FE	Yes	Yes	Yes	Yes	Yes	Yes	Yes	Yes	Yes	Yes
Bank controls	Yes	Yes	Yes	Yes	Yes	Yes	Yes	Yes	Yes	Yes
Macro controls	Yes	Yes	Yes	Yes	Yes	Yes	Yes	Yes	Yes	Yes
Other firm controls	No	Yes	No	Yes	No	Yes	No	Yes	No	Yes

The dependent variable in columns 1–10 is the probability of being credit constrained. MP_*t* − 2_ is the 1-year lag (equivalent to two survey waves) of the logarithm of the assets of individual central bank balance sheets—minus the autonomous factors—for stressed countries. Profit decreased, credit history deteriorated, own outlook deteriorated and own capital deteriorated are all categorical variables which proxy firm risk from the firm’s viewpoint. Innovation is a categorical variable which proxies if the firm innovated in the previous 6 months, and is a measure of firm risk. Country sector fixed effects, time sector fixed effects, bank controls and macro controls (both lagged by 1 year, equivalent to two survey waves) are included in all specifications. Firm controls are added in columns 2, 4, 6, 8 and 10. Robust standard errors are in parentheses.

*mn* million.

*Significance at 10%

**Significance at 5%

***Significance at 1%

The ECB deposit facility rate fell to zero in July 2012 and turned negative for the first time in June 2014 with negative rates being paid on banks’ reserves lodged with the ECB. This acted as an incentive for banks to lend to the real economy rather than to earn negative interest rates on their reserves. While banks keep a proportion of their increased deposits to meet their reserve, liquidity and capital requirements, the negative ECB deposit facility rate encourages lending and accentuates the effect of asset purchases on credit supply by increasing the cost of holding the reserves injected via asset purchases, thus incentivising banks to rebalance towards bank loans (Altavilla et al., 2019).^3^

The ECB then implemented a series of TLTROs, the first on 5 June 2014, the second (TLTRO II) on 10 March 2016 and a third series (TLTRO III) on 7 March 2019 (Bańkowska et al., 2020).^4^ TLTROs are Eurosystem operations that provide cheap financing to credit institutions and are designed to incentivise lending to SMEs. The more banks lend to SMEs, the lower the interest rate they pay to the ECB and so the lower the interest rate they can charge to SMEs. The TLTROs, therefore, reinforce the ECB’s accommodative policy stance and strengthen the transmission of monetary policy by further incentivising bank lending to the real economy.

In October 2014, the ECB announced an expanded APPs to promote lending to the real economy and increase inflation. This can occur directly, with institutions selling bonds to the ECB and using the proceeds to extend credit to the real economy or indirectly through banks’ lending of a proportion of their increased deposits, from investors who have sold bonds to the ECB and lodged the proceeds in their bank accounts, to firms (Cawley & Finnegan, 2019; ECB, 2015). The increased ECB demand for asset purchases also increases prices for sovereign bonds and pushes down their yields, leading to lower interest rates across the economy (Andrade et al., 2016; Eser et al., 2019; Larkin et al., 2019). The lower yields make it cheaper for firms to borrow externally and reduce the firm-specific user cost of capital, allowing them to invest more.

Table 1 shows that the above policy tools were employed throughout our sample of study, and hence instead of focusing on a particular non-standard monetary policy measure, we provide a general overview of the impact of UMP by exploiting the time series of UMP measures taken by the total assets of national central banks of each country. This measure reflects the series of UMP tools undertaken by the ECB that provided liquidity after the GFC and sovereign debt crisis.^5^ Further, the ECB uses central bank assets as a measure of monetary policy itself (ECB, 2015) and the literature generally measures UMP by using the total value of central bank assets (Boeckx et al., 2017; Gambacorta et al., 2014; Horvath et al., 2018; Peydró et al., 2021; von Borstel et al., 2016). The next section outlines the literature on the impact of monetary policy on credit constraints on firms in stressed countries and develops our two main hypotheses.

## Literature review and hypothesis development

This section focuses on the theoretical framework that underpins firms’ access to finance in an environment of UMP and develops our two hypotheses, which considers the interaction of UMP and firm-level heterogeneity in terms of firm leverage and firm risk.

### Monetary policy and firm leverage

There is a large body of theoretical and empirical evidence suggesting that access to finance for firms depends on the strength of their balance sheets (Jimenez et al., 2012; Holton et al., 2013; Blanco and Jimenez, 2018; McQuinn, 2019). For instance, Berger and Udell (1998), Masiak et al. (2019) and De Jonghe et al. (2020) show that smaller and younger firms find it more difficult to access finance due to asymmetric information and increased bank screening costs. Further, leverage is a common variable to proxy balance sheet health (Blanco and Jimenez, 2018). In general, higher leverage decreases firms’ access to finance. The literature that focuses on leverage and SMEs’ access to bank credit using firm-based surveys such as SAFE shows that firms with higher debt-to-assets ratios are more likely to experience credit constraints (Ferrando & Mulier, 2015; Kaya & Masetti, 2018; McQuinn, 2019; Mrkaic & Öztürk, 2014). Corbisiero and Faccia (2019) find being located in a stressed country and being more leveraged are both associated with a higher probability of credit rejection.

More recently, there is growing literature on the effects of monetary policy shocks on firm-level activity identified through firm heterogeneity. Cloyne et al. (2018) find evidence that monetary policy shocks are more sensitive to younger firms that pay no dividends. Jeenas (2019) show that high-leveraged firms with low liquid assets react strongly to fixed capital formation, inventories and sales growth as a result of monetary policy tightening, while Bahaj et al. (2019) find larger employment responses to monetary policy shocks for younger and more leveraged firms. Caglio (2021) using firm-bank loan level data from the USA finds that when monetary policy is expansionary, credit access of SMEs with high leverage increases more as their borrowing capacity expands, given their frequent use of earnings and operations-based collateral. Inspired by the recent studies, our paper identifies leverage as an observable characteristic of firms’ balance sheet strength and documents firms’ reactions to be credit constrained to monetary policy shocks.^6^ We hypothesize:

- Hypothesis 1 (H1): UMP decreases the probability of firms with increased debt-to-assets ratio being credit constrained.

It is expected that UMP for leveraged firms should make accessing bank finance easier via their improved balance sheets and collateral and that this should translate into a reduction in credit constraints. A negative relationship is, therefore, expected between the probability that a firm is credit constrained and the interaction between increased debt-to-assets ratio and UMP.

### Monetary policy and firm risk

A related channel of monetary policy transmission is the risk-taking channel which describes how UMP can lead to excessive risk taking (Borio & Zhu, 2012). For example, central bank asset purchases increase asset prices across a range of assets and decrease their yields, making lending to firms more attractive for banks in their search for yield (Kapoor & Velic, 2022). The low interest rate environment generated by UMP may encourage banks in their search for higher yield to extend credit to relatively risky firms (Andreeva & García-Posada, 2021; Jimenez et al., 2014; Jiménez et al., 2018; Maddaloni & Peydro, 2013). As a counterpart to the risk-taking literature of monetary policy, we hypothesize:

- Hypothesis 2 (H2): UMP reduces the probability of risky firms being credit constrained.

It is expected that given the low-interest rate environment generated by UMP, banks will chase higher yields and that this will manifest itself in lending to riskier firms. We use a future predictor of risk, profit decreased in the previous 6 months as well as a selection of subjective measures of risk such as firms’ own view if their credit history, own economic outlook or own capital has deteriorated in the previous 6 months and, finally, an activity-based measure of risk, i.e. innovative activity, given that such activity is more uncertain and, therefore, riskier. A negative relationship between firms’ credit constraints and the interaction of firm risk and UMP is expected.

### Stressed versus non-stressed countries

The literature shows that firms in stressed countries relative to non-stressed countries were more credit constrained following the financial and sovereign crises from 2008 to 2013 due to, inter alia, higher bank funding costs, higher firm leverage, poorer macroeconomic performance and deteriorated bank balance sheets—which made banks more risk averse—in stressed countries. Therefore, H1 and H2 are tested for both stressed and non-stressed countries over the period 2014 to 2019 when UMP increased in intensity in order to provide a full comparison. Non-stressed countries include Austria, Belgium, Finland, France, Germany and the Netherlands—those countries that did not experience elevated average sovereign yields during the 2008–2013 period—and this definition follows the literature (Ferrando et al., 2017 and Kaya & Masetti, 2018).

## Data and empirical methodology

We employ biannual firm-level data from the ECB/EC SAFE for our analysis. There have been twenty-seven SAFE waves conducted since 2009, the period when the financial crisis infected the euro area. Our data covers the period 2014:H1–2019:H1 for five stressed countries, i.e. Greece, Ireland, Italy, Portugal and Spain. Our choice of starting date of the sample includes the introduction of negative policy rates (June 2014), the first series of TLTROs (September 2014) and the ECB’s announcement of the APPs (October 2014). We omit observations with missing values for credit constrained. We further remove all firms that have more than 250 employees or a turnover exceeding EUR 50 million, so that our final sample contains only SMEs according to the definition applied by the European Commission (2003). This leaves us with a final sample of 11,319 observations.

The firm-level SAFE includes information on non-financial firms’ responses to questions regarding their characteristics in terms of size, age, legal form, ownership and sector. Further, it includes information on whether firms increased their debt-to-assets ratio or profit in the previous 6 months. It includes the firm’s own assessment of their credit risk and whether they engaged in innovative activity in the previous 6 months.^7^ All survey-based percentages are weighted statistics that restore the proportions of the economic weight (in terms of employees) of each class size, economic activity and country (ECB, 2022b). We use this data to construct our dependent as well as firm-level explanatory variables. The information in SAFE is qualitative, and so, all firm-level variables are categorical. Definitions of all our variables can be seen in Table 7 in Appendix 2.

Following the literature on financial constraints (Bańkowska et al., 2020; Corbisiero & Faccia, 2019; Ferrando et al., 2017; Kaya & Masetti, 2018) and the relevance of bank loans to SMEs in our sample (92.7%), we construct our dependent variable using firm responses to Q7a and Q7b on firms’ credit access. Q7a and Q7b from the SAFE can be seen in Table 6 of Appendix 1. The dependent variable ‘credit constrained’ is equal to 1 if the firm reported to have (i) applied for bank loans in the previous 6 months but was rejected (credit denied) or (ii) applied but received less than 75% of its demand (rationed) or (iii) refused credit because it was offered at too high a cost (refused due to high cost) or (iv) not applied because of possible rejection (discouraged). Alternatively, the variable is equal to 0 if the firm reported to have applied for bank loans in the previous 6 months and received everything or received 75% and above.

**Fig. 1:**
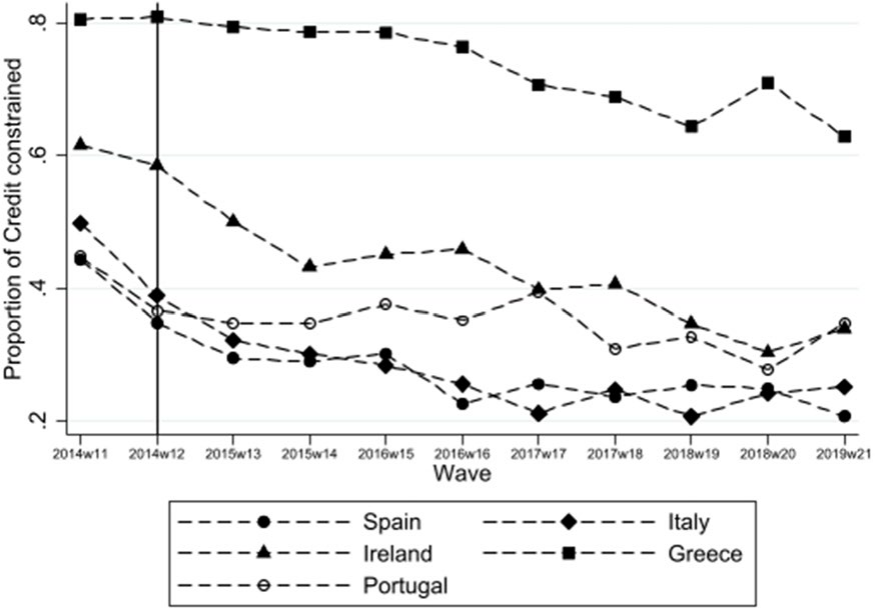
Proportion of credit constrained by country from 2014 to 2019 for stressed countries. The vertical line indicates the beginning of UMP in the form of negative interest rate policy, forward guidance, TLTROs and APPS

The inclusion of discouraged borrowers is a major advantage of SAFE over credit registers data, which has been used widely to measure loan supply. In particular, it captures informal credit constraints such as discouraged firms and disregarding discouraged borrowers would risk measurement error in the supply of credit (Ferrando et al., 2019; McQuinn, 2019). Of the 11,319 firms in our sample, 4581 or 40.5% were credit constrained—with discouraged borrowers making up 40% of these firms.^8^ Figure 1 shows the proportion of credit-constrained firms in each country over the period 2014 to 2019. It shows that while credit-constrained firms remain elevated in Greece, the proportion of credit-constrained firms fall over the period 2014–2019, which is happening at the same time as UMP is increasing in intensity.

Next, we follow Peydró et al. (2021) to measure monetary policy by the total assets of the ECB minus the autonomous factors that are outside the direct control of the ECB—such as banknotes in circulation and government balances at the central bank. In order to provide an appropriate guide for the implementation of APPs across euro-area countries, the ECB’s capital key was considered the most appropriate metric since it is based on the population and the size of the economy in each country (Larkin et al., 2019). Therefore, we employ data from the ECB and construct a variable from individual country central bank balance sheets. Table 4 provides summary statistics for the monetary policy indicator and shows that the average assets purchased in the stressed countries was €0.194 trillion, ranging from €0.00573 trillion to €0.335 trillion over the period 2014 to 2019. In a robustness check, we also use monetary policy measures at the country level following Horvath et al. (2018).

To test *H1*, we model Eq. 1 as follows:

1 $$P\left({\mathrm{CreditConstrained}}_{i,c,t}=1|x\right)={\alpha }_{i}+{\beta }^{\mathrm{^{\prime}}}{\mathrm{MP}}_{c,t-2}+{\gamma }^{\mathrm{^{\prime}}}{\mathrm{DebttoAssets}}_{i,c,t}\times {\mathrm{MP}}_{c,t-2}+{\delta }^{\mathrm{^{\prime}}}{X}_{i,c,t}+{\theta }^{\mathrm{^{\prime}}}{\mathrm{Macro}}_{c,t-2}+{\varphi }^{\mathrm{^{\prime}}}{\mathrm{BankCh}}_{c,t-2}+{\tau }_{c,s}+{\varepsilon }_{i,s,c,t}$$

where $P\left(\mathrm{CreditConstrained}=1|x\right)$ is a dummy variable equal to 1 for credit-constrained firm $i$ in country $c$ in half-year $t$, and 0 otherwise. ${\mathrm{MP}}_{t-2}$ is our measure of monetary policy and is proxied by a 1-year (equivalent to two survey waves) lag of the logarithm of total assets of the individual country central bank balance sheet minus the autonomous factors. We take the 1-year lag since monetary policy can impact firms’ decisions to apply for finance and banks’ decisions to grant loans with a lag. ${\mathrm{DebttoAssets}}_{i,c,t}$ is a binary variable equal to 1 if the firm’s debt-to-assets ratio increased in the previous 6 months, and 0 if it decreased or remained unchanged. Table 2 highlights that 30% of firms experienced an increased debt-to-assets ratio.

${\gamma }^{\mathrm{^{\prime}}}$ is our main coefficient of interest that captures if firms with increased debt-to-assets ratio decreased their probability of being credit constrained during UMP. A statistically significant and negative (positive) result of the interaction between monetary policy and firms’ increased debt-to-assets ratio would suggest that the implementation of UMP reduces (increases) the probability of firms being credit constrained.

We add in time fixed effects when monetary policy is measured at the country level to exclude unobserved variables that evolve over time but are constant across firms. Finally, we include sector-country fixed effects ${(\tau }_{c,s})$ to eliminate any shocks common to all firms in the same sector and in the same country. Since our panel is an unbalanced one with some firms being interviewed once, this limits our use of firm fixed effects which would further allow isolating the impact of UMP on firm credit constraints by absorbing any firm-specific credit demand shocks.

We control for confounding factors that might influence loan supply and loan demand such as bank characteristics, firm level heterogeneity and the stage of the economic cycle. ${\mathrm{BankCh}}_{c,t-2}$ measures banks’ balance sheet health indicators at the country level which impacts credit supply and demand. ${X}_{i,c,t}$ is a set of firm-level covariates to control for firm heterogeneity with subscripts $i$, $c$ and $t$ indicating firm, country and time, respectively. ${\mathrm{Macro}}_{c,t-2}$ is a vector of macroeconomic variables to control for the economic cycle. Both macro and bank controls are lagged 1 year, in line with Mc Namara et al. (2020) that lagged their explanatory variables 1 year to minimise endogeneity concerns.

We justify our controls in line with the literature. The use of non-performing loans (NPL)-to-total loans and capital ratios at the country level is well highlighted in the empirical evidence that the transmission of monetary policy to the real economy is based primarily on bank balance sheet health. In particular, the loan supply may be impaired for banks with high NPLs or weak capital (Altavilla et al., 2019; Donnery et al., 2018). Table 4 shows NPLs vary from a low rate of 3.4% to a high rate of 45.8%, while capital ratios vary from a low rate of 10.6% to a high rate of 25.2% over the period. We also control for the macroeconomic cycle using unemployment and inflation, given the country-specific macroeconomic impact on firms’ access to finance directly and indirectly (Ferrando & Ganoulis, 2020).

We include firm characteristics using information on firm size (proxied by employees and turnover) and age. Further, we include legal form, in particular, whether firms are stand-alone—autonomous enterprises making independent financial decisions. We expect stand-alone firms to be less credit constrained as they may be less discouraged from applying for finance because they have a higher need for external financing (Freel et al., 2012; Mol-Gómez-Vázquez et al., 2019). In addition, we include ownership. Family firms usually have more durable banking relationships in comparison to non-family firms reducing asymmetric information (Calabrese et al., 2021). Therefore, we expect family-owned firms to be less credit constrained.

Finally, we include sector,^9^ given that firms in different sectors also have different financing needs (Moritz et al., 2016). For example, firms in the services sector might require less external financing due to lower capital requirements or firms in the industry sector may require more long-term financing due to their larger share of long-term assets (Masiak et al., 2019). Firms in industry may also be less credit constrained as they have more fixed assets which increase collateral, and this reduces risks for banks’ lending to firms (Guercio et al., 2020; Moritz et al., 2016). Further, firms in different sectors may react differently to monetary policy (Durante et al., 2020).

Table 4 shows the summary statistics on all the firm variables included in the survey and employed in our tests. As averages over the sample, 40% of total firms are micro (less than 10 employees), 33% are small (between 10 and 50 employees) and 27% are medium (between 50 and 250 employees). Almost 54% of firms have an annual turnover of less than €2 million.^10^ Most firms (86%) are more than 10 years old, with only 0.8% less than 2 years old; 94% of the firms are stand-alone while 87% are individual or family owned; 58% are in industry while 33% are in trade, while 9% are in construction.

To test *H2*, we model Eq. 2 as follows:

2 $$P\left({\mathrm{CreditConstrained}}_{i,c,t}=1|x\right)={\alpha }_{i}+{\beta }^{\mathrm{^{\prime}}}{\mathrm{MP}}_{c,t-2}+{\gamma }^{\mathrm{^{\prime}}}{\mathrm{FirmRisk}}_{i,c,t}\times {\mathrm{MP}}_{c,t-2}+{\delta }^{\mathrm{^{\prime}}}{X}_{i,c,t}+{\theta }^{\mathrm{^{\prime}}}{\mathrm{Macro}}_{c,t-2}+{\varphi }^{\mathrm{^{\prime}}}{\mathrm{BankCh}}_{c,t-2}+{\tau }_{c,s}+{\varepsilon }_{i,s,c,t}$$

The interaction between monetary policy and firm risk variables is the main coefficient of interest as it captures the probability of a risky firm being credit constrained during periods of UMP. We model ${\mathrm{FirmRisk}}_{i,c,t}$ categorically, and the measures for firm risk include a future predictor of risk—profit decreased in the previous 6 months, as well as a selection of subjective measures of risk—firms’ own view if there has been deterioration in credit history, own economic outlook and own capital in the previous 6 months and, finally, an activity-based measure of risk innovative activity, given that such activity is more uncertain and, therefore, riskier. Table 2 shows that 40% of firms experience a reduction in profit in the last 6 months; 15% of firms report their credit history deteriorated; 31% firms witness a deterioration in their own outlook in the previous 6 months, while 15% report their own capital deteriorated. Further, 33% of firms had engaged in some sort of innovation in the previous 6 months.

The literature shows that decreased profits increase the probability of a firm being credit constrained (Beyhaghi et al., 2020; Ferrando & Mulier, 2015; Holton et al., 2014). In addition, a deterioration in a firms’ own view of their credit history, own economic outlook and own capital should reduce their access to finance (Aristei & Gallo, 2022; Calabrese et al., 2021; Moro et al., 2020). The literature also indicates that firms that engage in innovation face more credit constraints due to the uncertainty associated with this activity (Acharya & Xu, 2017; Bańkowska et al., 2020; Brown et al., 2022; Guercio et al., 2019; Moro et al., 2020; Santos & Cincera, 2022). A statistically significant and negative (positive) result in the interaction of monetary policy and firm risk would suggest that the implementation of UMP reduces (increases) the probability of risky firms being credit constrained.

## Results

This section discusses the impact of UMP on the probability of a firm being credit constrained. First, we consider the role of debt-to-assets ratio in determining whether firms are less credit constrained. Second, we present results pertaining to firm-level indicators of risk. Lastly, we present a sub-sample analysis followed by a series of robustness checks for our main results.

### Impact of UMP on firms’ probability of being credit constrained: role of firm leverage

Table 3 presents the estimation results for Eq. 1 assessing the impact of implementation of UMP on the probability that a leveraged firm will be credit constrained. Columns 1–4 show the results for stressed countries while columns 5–8 report results for non-stressed countries. The dependent variable in columns 1–8 is the probability of being credit constrained. MP_*t* − 2_ is 1-year lag (equivalent to two survey waves). Reported estimates are conditional marginal effects drawn from probit regression models with sample selection for our pooled sample of SMEs.

Columns 1–4 show that when we interact monetary policy with increased debt-to-assets ratio over the previous 6 months for stressed countries, we find that firms are more likely to be credit constrained and this is statistically significant at the 1% level across all specifications. The implementation of UMP increases the probability of firms with increased debt-to-assets ratio being credit constrained. Here, increased debt-to-assets ratio may indicate that the firm is riskier and, therefore, they did not benefit from UMP over the period 2014–2019. This is in line with the literature on the determinants of firm’s access to finance that shows a negative relationship between leverage and credit constraints as leverage is often used as an inverse proxy of firm credit quality (Ferrando & Mulier, 2015; Kaya & Masetti, 2018; McQuinn, 2019; Mrkaic & Öztürk, 2014). In theory, the higher the debt ratio, the greater the degree of financial risk because more levered firms, everything else equal, face a greater likelihood of insolvency (Demoussis et al., 2017). This may be because a firm with higher leverage is more likely to default as they need higher profits to be able to repay their debt.

Regarding firm-level controls, our results also suggest smaller and younger firms are more likely to be credit constrained. Further, we find that stand-alone firms that make independent financial decisions are less likely to be credit constrained and discouraged because they have a higher need for external financing and this finding is reflected in the literature (Freel et al., 2012; Mol-Gómez-Vázquez et al., 2019). In terms of sector, the only significant result is for industry. Firms in industry are less likely to be credit constrained as they are more likely to be financed by debt (Moritz et al., 2016), show the lowest incidence of credit constraints (García-Posada Gómez, 2019) and are more likely to apply for and obtain bank loans relative to the trade or services sector (Guercio et al., 2020).

In contrast to our findings for SMEs in stressed countries, columns 5–8 show results for firms located in non-stressed countries. We find negative but statistically insignificant coefficients of the interaction term between monetary policy and increased debt-to-assets ratio over the previous 6 months. The impact of higher indebtedness on SMEs’ credit constraints was more pronounced in stressed countries in the aftermath of the financial and sovereign debt crisis (Fernández de Guevara et al., 2021). Corbisiero and Faccia (2019) find that being located in a stressed country and being more leveraged are both associated with a higher probability of credit rejection. This may be because banks are more sensitive to leveraged firms when extending credit in stressed countries due to the fall-out from their excessive growth in credit prior to the financial crisis and its consequent repercussions for banks (Cussen & O'Leary, 2013; Fernández de Guevara et al., 2021) and tighter regulatory requirements in stressed countries (Altavilla et al., 2019). Our findings suggest that this heterogeneity may still be at play over the period 2014–2019 when UMP increased in intensity. This is important, as UMP may filter down to SMEs unevenly depending on where they are located, and this could have distributional consequences.

### Impact of UMP on firms’ probability of being credit constrained: role of firm risk

Table 4 presents the estimation results for Eq. 2 showing the impact of UMP interacted with the firms’ risk variables for stressed countries, while Table 5 presents the results for non-stressed countries. In both tables, columns 2, 4, 6, 8 and 10 control for firm-level characteristics, while the results in other columns pertain to only bank and macro-level controls. All the interaction terms of firm risk—from the firms’ viewpoint—interacted with MP_*t* − 2_ are insignificant in impacting on firms’ credit constraints, and this result holds for firms in both stressed and non-stressed countries.

The risk-taking channel literature generally focuses on how monetary policy affects the portfolio rebalancing in bank balance sheets away from safe assets like government bonds and towards more risky assets like loans to SMEs (Albertazzi et al., 2021). When we measure risk from the firms’ viewpoint, we find no significant relationship between UMP and the decreased probability that such risky firms are credit constrained. Dell'ariccia et al. (2017), Burlon et al. (2019), Ertan et al. (2020), Peydró et al. (2021) and Betz and De Santis (2022) find little evidence of risk-taking arising from bank behaviour due to UMP. Our paper complements this area of research, suggesting that there is little evidence of risky firms being granted loans when risk is measured from the firm’s viewpoint.

### Sub-sample analysis

**Fig. 2:**
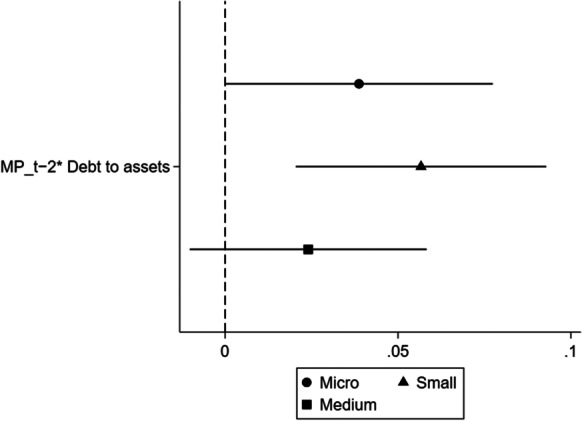
Hypothesis 1, by size. A dependent variable is a probability of a firm being credit constrained

**Fig. 3:**
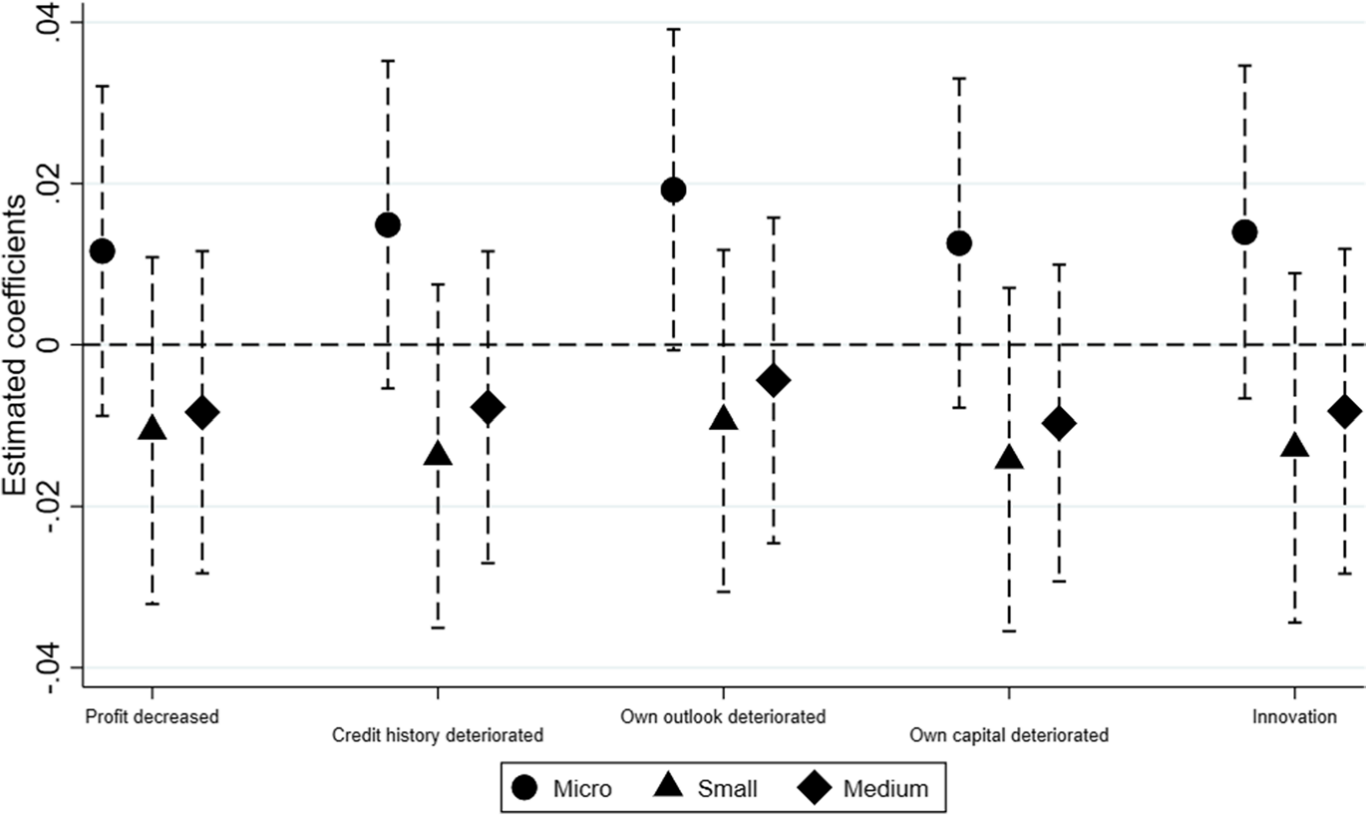
Hypothesis 2 (${MP}_{t-2} \times {FirmRisk}_{i,c,t})$, by size. A dependent variable is a probability of a firm being credit constrained

**Fig. 4:**
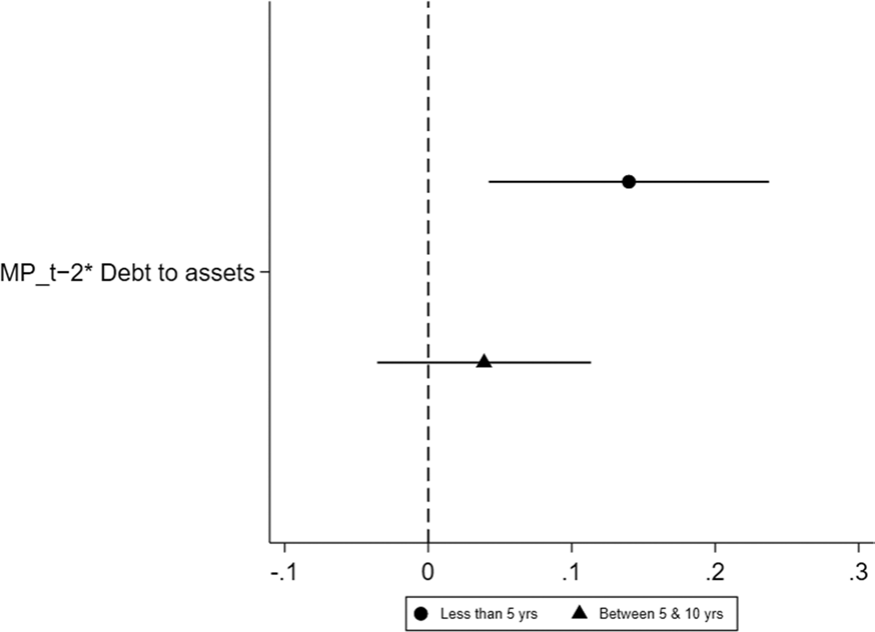
Hypothesis 1, by age. A dependent variable is a probability of a firm being credit constrained

**Fig. 5:**
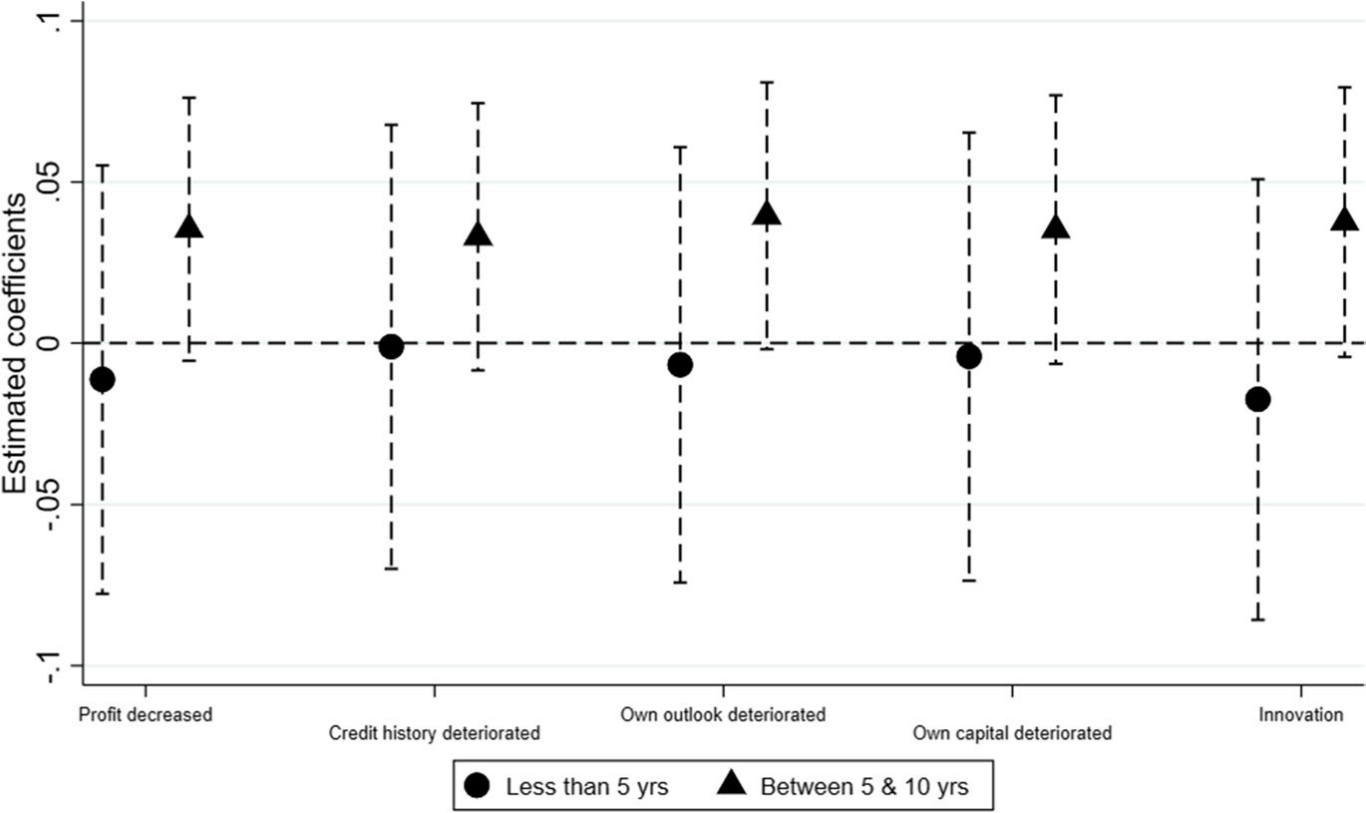
Hypothesis 2 (${MP}_{t-2} \times {FirmRisk}_{i,c,t}$), by age. A dependent variable is a probability of a firm being credit constrained

The results suggest that there is a possibility of heterogeneity related to size or age in our sample. Previous literature suggests that younger and smaller firms have higher financial constraints due to their inherent risks (Berger & Udell, 1998; Bernanke et al., 1996; Artola & Genre, 2011; Ferrando & Mulier, 2015). To investigate the impact of UMP on SMEs’ access to finance based on firm size and age, we run Eqs. 1 and 2 where we categorise firms, first, as micro, small and medium (Figs. 2 and 3) and, second, as young and old (Figs. 4 and 5).

#### By size: proxied by employees^11^

This section defines firms as micro, small and medium based on the number of employees, where firms with less than 10 employees are defined as micro, firms with employees between 10 and 50 are small, while firms with employees between 50 and 250 are medium. Figure 2 plots the coefficients for credit constrained regressed on debt-to-assets ratio increased during periods of UMP. It shows that ${\mathrm{MP}}_{t-2} \times {\mathrm{Debttoassets}}_{i,c,t}$ is positive and statistically significant for micro and small firms, confirming that these firms are credit constrained. We do not find any evidence for medium firms. For Fig. 3, all firm risk variables interacted with monetary policy are statistically insignificant for micro, small and medium firms, implying that we find no evidence that banks are lending to risky firms.

#### By age

Next, we turn to SMEs characterised as young and old. Young firms are the ones that are less than 5 years of age, while old firms are between 5 and 10 years of age.^12^ Figures 4 and 5 plot the coefficients for credit constrained for hypotheses 1 and 2, respectively. Figure 4 shows that ${\mathrm{MP}}_{t-2} \times {\mathrm{Debttoassets}}_{i,c,t}$ is positive and statistically significant at the 5% level for young firms, while the coefficient is statistically insignificant for firms over 5 years old. This confirms that the implementation of UMP increases the probability of younger firms being credit constrained. In Fig. 5, all coefficients are statistically insignificant for firms regardless of their age. This confirms our results for H2 that there is no evidence that banks are lending to risky firms during periods of UMP, when risk is measured using firm-level variables.

### Robustness checks

#### Robustness by type of credit constraint

Including discouraged firms in our dependent variable could lead to overestimation, given that discouraged borrowers make up 40% of credit-constrained firms in our sample. Therefore, we first take discouraged borrowers only (Table 8 in Appendix 3, column 1) and then credit constrained, excluding discouraged borrowers (Table 8 in Appendix 3, column 2) to see if our *H1* and *H2* results are driven by this definition. Our results show that the implementation of UMP increases the probability of firms with increased debt-to-assets ratio being credit constrained, whether or not we include discouraged borrowers in our dependent variable. Table 9 in Appendix 3 takes discouraged borrowers only as the dependent variable, and the results are similar to our main findings for *H2* in Table 4.

#### Robustness by type of central bank asset

Central banks’ total assets might be considered a too broad proxy for UMP, given that central bank asset purchases did not directly impact SME credit constraints. This is because asset purchases mainly benefit large companies by increasing the demand for their bonds and pushing down their yields (Albertazzi et al., 2021). Indeed, in our sample, 90% of firms indicated that they did not issue debt securities in the past or considered doing so in the future.^13^

We therefore construct two further measures of country-level monetary policy in stressed countries following Horvath et al. (2018). First, we use a measure which uses data on holdings of government debt securities only. Second, we use a broader measure of the overall measure of the scope of the ECB’s balance sheet policy calculated as the sum of holdings of government debt securities, debt securities issued by Monetary Financial Institutions (MFIs) and loans to MFIs.

The results for *H1* are presented in Table 10 in Appendix 3 and are quantitatively similar to the ones obtained in our main specification in Table 3. For *H2*, Table 11 in Appendix 3 uses holdings of government debt securities as the dependent variable. Overall, the results are similar to the main findings in Table 3, with one exception—firms’ own economic outlook. The implementation of UMP leads to firms whose own economic outlook deteriorated in the previous 6 months to be more credit constrained. Next, Table 12 in Appendix 3 employs the sum of holdings of government debt securities, debt securities issued by MFI and loans to MFI as our dependent variable to test *H2*. The interaction term between monetary policy indicator and firm’s own credit history is statistically significant, implying implementation of UMP leads to firms whose own credit history deteriorated in the previous 6 months to be more credit constrained. However, on balance, the interaction coefficients between the two additional measures of monetary policy and the rest of the risk variables are statistically insignificant.

## Discussion and conclusions

We examine the relationship between UMP and SME access to finance in stressed countries over the period 2014–2019 when UMP increased in size and scope. We employ firm micro-level data from the EU/ECB’s SAFE. Using a binary probit model, our results suggest that in the presence of UMP, firms with higher debt-to-assets ratio are credit constrained in stressed countries and this finding is robust to different definitions of credit constrained and type of central bank assets. We could argue that, in line with the literature, firms with higher debt-to-assets ratio are riskier and would find it costly to access new debt; hence, the probability of them being credit constrained increases and this effect remains even in the presence of UMP. However, this finding does not hold for non-stressed countries. We could argue that banks in stressed countries are more sensitive to higher leveraged firms when extending credit, even in an environment of UMP over the period 2014–2019. This could be due to the legacy of the negative fallout for banks in stressed countries from taking on excessive risk prior to the financial crisis (Blanco and Jimenez, 2018; Corbisiero & Faccia, 2019; Fernández de Guevara et al., 2021) and the tighter regulatory environment for banks in stressed countries following the two crises (Altavilla et al., 2019).

Further, we find no evidence that the implementation of UMP reduces the probability of risky firms being credit constrained, when risk is measured using firm-level variables. This finding is also robust to different definitions of credit constraint and type of central bank asset. We also conduct a sub-sample analysis, where we show that firm size and age play a crucial role in shaping differences in external financing conditions for SMEs in stressed countries. This is in line with the literature that suggests smaller and younger firms are a proxy for riskier firms and are, therefore, more likely to be credit constrained (Berger & Udell, 1998; Bernanke et al., 1996; De Jonghe et al., 2020; Masiak et al., 2019). This is mainly due to information asymmetries between lenders and borrowers, given the opacity of smaller and younger firms (Berger & Udell, 1998) which makes them inherently riskier (Bernanke et al., 1996; Cole & Sokolyk, 2016; Ferrando et al., 2017; Guiso & Minetti, 2010).

The transmission of UMP to SMEs is vital, given their bank dependence and importance in terms of economic activity, and there are a number of policy implications arising from this research. First, public policy could intervene to support banks to become more digitalised to reduce asymmetric information between lenders and small and young borrowers. For example, blockchain technology could monitor the flow of transactions from banks to firms on the supply chain of finance in real time. This would reduce screening costs for banks enabling them to assess the risk profile of smaller and younger firms and support their ability to access finance. Further, monetary policy should operate in a manner that SMEs in all euro-area countries have similar access to bank finance. Increased digitalisation in the banking sector would allow banks to be better informed about the risk profile of leveraged firms and allocate funding to SMEs that warrant liquidity to increase investment and growth. Second, we show that the implementation of UMP increases the probability that firms with increased debt-to-assets ratio are discouraged from applying for a loan for fear of rejection. Public policy could also intervene to educate firms about the increased willingness of banks to lend to SMEs during periods of expansionary or unconventional monetary policy.

Fourth, the literature shows that SMEs use a limited number of sources of finance (Bańkowska et al., 2020; Cressy & Olofsson, 1997). This pattern is confirmed in our sample of SMEs.^14^ The high persistence in firms’ demand for bank financing is motivated by the limited recourse to capital markets and by the role of application costs (Aristei & Angori, 2022). The development of sustainable and diversified financing for micro firms other than banks is also important to support small and young firms’ growth. Diversification across alternative financing instruments can make an important contribution to resilience against adverse financial and real shocks. The diversification of funding options for SMEs as one of the Capital Markets Union’s (CMU’s) priorities is to be welcomed in this regard. While there have been efforts since 2015 to develop a CMU (Bańkowska et al., 2020), policy should now focus on implementing this initiative to facilitate SMEs’ fundraising.

This is even more important as we enter a phase of SME government support tapering in a post-pandemic environment and ECB monetary policy tightening in an inflationary environment. SME credit access was supported throughout the pandemic via extraordinary emergency government supports and creditor forbearance.^15^ The tapering of government supports may result in a rise in credit demand over the coming months. In addition, the ECB has tightened monetary policy and signalled further tightening to grapple with high inflation (ECB, 2022a). Monetary policy will need to channel funds to those SMEs that survived the pandemic and can grow. McCann et al. (2021) show that adequate availability of liquidity finance remains a key priority for facilitating SME recovery post pandemic. It is important to ensure that the phase out of emergency support and monetary policy tightening does not create an SME solvency crisis, especially for those firms that incurred increased debt-to-assets ratios to survive the pandemic.

There is room for more work. First, we provide a general overview of the impact of UMP by exploiting the time series of UMP measures taken by the total assets of national central banks of each country. Future research could isolate the impact of various monetary policy tools on leveraged SMEs’ access to finance. Second, as we have seen from the literature, innovation is an important measure when considering the probability of a firm being credit constrained. Considering if SMEs change innovation activity on account of being more credit constrained or not is an avenue for future research. Third, for our analysis, we use micro firm-level data from SAFE. In order to have a clear understanding on how banks use additional funds provided by the ECB and whether this is transmitted to the real economy, a potential extension could match firm-bank observations and investigate which banks lent to which firms. Further, it could be assessed at what level of debt-to-assets ratio does UMP increase the probability that SMEs will be credit constrained. Fourth, we show leveraged firms are relatively more credit constrained in stressed countries even during UMP. Further research could also investigate the reasons for this heterogeneity and devise policy responses to ensure a more even transmission of monetary policy across countries. This takes on more relevance in the context of rising ECB rates and inflation, which could further aggravate the uneven transmission of monetary policy across the euro area. Indeed, this asymmetric transmission of monetary policy is in the forefront of policy-makers’ minds as signalled by the ECB’s Transmission Protection Instrument (TFI) announced in July 2022 (ECB, 2022c), to ensure that the monetary policy stance is transmitted smoothly across all euro-area countries.

## Figures and Tables

**Fig. 6:**
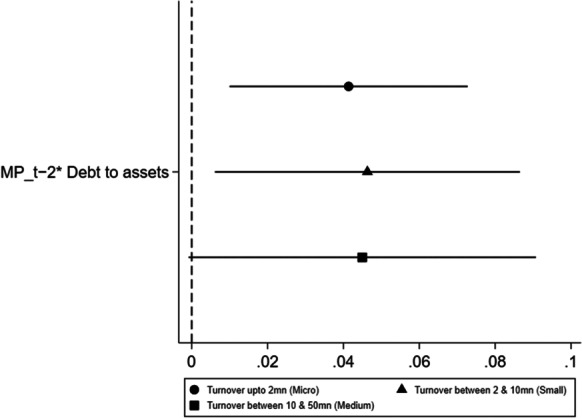
Hypothesis 1, by size, proxied by turnover. A dependent variable is a probability of a firm being credit constrained

**Fig. 7:**
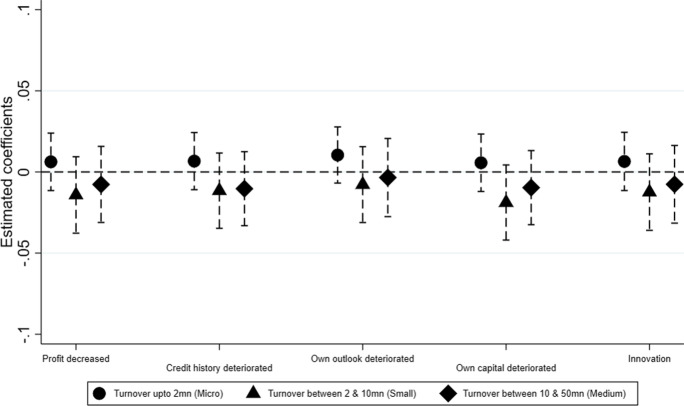
Hypothesis 2 (MP_*t* − 2_ × FirmRisk_*i*,*c*,*t*_), by size, proxied by turnover. A dependent variable is a probability of a firm being credit constrained

## Data Availability

The data can be obtained from the official websites used: ECB/EC Survey on the access to finance of enterprises (SAFE) https://www.ecb.europa.eu/stats/ecb_surveys/safe/html/index.en.html ECB Statistical Data Warehouse, https://sdw.ecb.europa.eu/, Eurostat, https://ec.europa.eu/eurostat IMF Financial Soundness Indicators, https://data.imf.org/.

## Appendix 1

Table 6

**Table 6:** Dependent variable questionnaire

Survey question	Survey answer
Q7A: Have you applied for a bank in the past 6 months? Please provide a separate answer in each case	• Applied • Did not apply because of possible rejection • Did not apply because of sufficient internal funds • Did not apply for other reason • Don’t know
Q7B: If you applied and tried to negotiate a bank loan over the past 6 months, what was the outcome? Please provide a separate answer in each case	• Received everything • Received 75% and above • Received below 75% • Refused because the cost was too high • Was rejected • Application is still pending • Don’t know

## Appendix 2

Variables employed: construction, source and corresponding definition.

Table 7

**Table 7:** Variable definition and source

Variable	Data source	Definition
***Dependent variable***		
Credit constrained	ECB/EC Survey on the Access to Finance of Enterprises (SAFE) Q7a, Q7b	Binary variable = 1 if the firm reported (i) to have applied for bank loans in the previous 6 months but was rejected (credit denied) or (ii) to have applied but received less than 75% of its demand (rationed) or (iii) to have refused credit because it was offered at a too high cost (refused due to high cost) or (iv) not to have applied because of possible rejection (discouraged). 0 = if the firm reported (i) to have applied for bank loans in the previous 6 months and they received everything or (ii) they received 75% or more of their demand
***Firm variables***		
Micro	ECB/EC SAFE QD1	= 1 if the firm has between 1 and 9 employees, 0 otherwise
Small	ECB/EC SAFE QD1	= 1 if the firm has between 10 and 49 employees, 0 otherwise
Medium	ECB/EC SAFE QD1	= 1 if the firm has between 50 and 249 employees, 0 otherwise
More than 10 years	ECB/EC SAFE QD5	= 1 if the firm is 10 + years old, 0 otherwise
Between 5 and 10 years	ECB/EC SAFE QD5	= 1 if the firm is between 5 and 10 years old, 0 otherwise
Between 2 and 5 years	ECB/EC SAFE QD5	= 1 if the firm is between 2 and 5 years old, 0 otherwise
Less than 2 years	ECB/EC SAFE QD5	= 1 if the firm is less than 2 years old, 0 otherwise
Stand-alone firm	ECB/EC SAFE QD2	= 1 if the firm is an autonomous profit-oriented enterprise, 0 otherwise
Individual or family owned	ECB/EC SAFE QD2	= 1 if the firm’s owner is an individual or a family, 0 otherwise
Turnover up to 2 mn	ECB/EC SAFE QD4	= 1 if the firm’s annual turnover is less than €2 mn, 0 otherwise
Turnover between 2 and 10 mn	ECB/EC SAFE QD4	= 1 if the firm’s annual turnover is between €2 mn and €10 mn, 0 otherwise
Turnover between 10 and 50 mn	ECB/EC SAFE QD4	= 1 if the firm’s annual turnover is between €10 mn and €50 mn, 0 otherwise
Turnover over 50 mn	ECB/EC SAFE QD4	= 1 if the firm’s annual turnover is €10 + mn, 0 otherwise
Industry	ECB/EC SAFE QD3	= 1 if the firm’s main activity is in industry, 0 otherwise
Construction	ECB/EC SAFE QD3	= 1 if the firm’s main activity is in construction, 0 otherwise
Wholesale or retail trade	ECB/EC SAFE QD3	= 1 if the firm’s main activity is in wholesale or retail trade, 0 otherwise
Services	ECB/EC SAFE QD3	= 1 if the firm’s main activity is services, 0 otherwise
Debt-to-assets ratio increased	ECB/EC SAFE Q2	= 1 if the firm’s debt-to-assets ratio increased in the past 6 months, 0 if it remained unchanged or decreased
***Firm credit risk variables***		
Profit decreased	ECB/EC SAFE Q2	= 1 if the firm’s profit decreased in the past 6 months, 0 if it remained unchanged or increased
Credit history deteriorated	ECB/EC SAFE Q11	= 1 if the firm’s credit history deteriorated in the previous 6 months, 0 if it remained unchanged or improved
Own capital deteriorated	ECB/EC SAFE Q11	= 1 if the firm’s own capital deteriorated in the previous 6 months, 0 if it remained unchanged or improved
Own outlook deteriorated	ECB/EC SAFE Q11	= 1 if the firm’s own outlook deteriorated in the previous 6 months, 0 if it remained unchanged or improved
Innovation	ECB/EC SAFE Q1	= 1 if the innovated (in terms of new or improved product, new or improved production process, new organisation of management, new way of selling goods or services) in the previous 6 months, 0 if did not
***Monetary policy variables for stressed countries***		
ECB BS assets (mn)	ECB Statistical Data Warehouse Central Statistics Office, Ireland	Continuous variable, monthly, total ECB assets (after subtracting the autonomous factors that are beyond the direct control of the ECB including banknotes in circulation and government balances) from individual central bank balance sheet for stressed countries, following Peydró et al. (2021). Monthly data averaged over half years ending in March and September
ECB government securities	ECB Statistical Data Warehouse	Continuous variable, monthly, measure of quantitative easing uses data on holdings of government debt securities from individual central bank balance sheet for stressed countries, following Horvath et al. (2018). Monthly data averaged over half years ending in March and September
ECB government and MFI securities and loans to MFIs	ECB Statistical Data Warehouse	Continuous variable, monthly; overall measure of the scope of balance sheet policies is calculated as the sum of holdings of government debt securities; debt securities issued by MFI and loans to MFI from individual central bank balance sheet for stressed countries following Horvath et al. (2018). Monthly data averaged over half years ending in March and September
***Macroeconomic variables***		
Unemployment	Eurostat	Continuous variable, unemployment rate (share of active population), seasonally adjusted. The unemployment rate is the number of people unemployed expressed as a share of the labour force. The labour force is the total number of people employed and unemployed. Quarterly data averaged over half years ending in March and September and expressed as decimals
Inflation	Eurostat	Continuous variable, inflation rate measured by HICP monthly data (annual rate of change). Average of monthly inflation rate data over half years ending in March and September and expressed as decimals
***Bank characteristics***		
Non-performing loans	IMF financial soundness indicators	Continuous variable, quarterly non-performing loans as a share of total gross loans averaged over half years ending in March and September and expressed as decimals
Regulatory capital to risk-weighted assets ratio	IMF financial soundness indicators	Continuous variable, quarterly regulatory tier 1 capital as a share of risk-weighted assets averaged over half years ending in March and September and expressed as decimals

*mn* million.

## Appendix 3

Figs. 6 and 7

Table 7, 8, 9, 10, 11, and 12

**Table 8:** H1 robustness by the type of credit constraint

Dependent variables	Discouraged	Credit constrained excluding discouraged
	1	2
MP_*t* − 2_	− 0.0245 (0.0252)	− 0.00707 (0.0312)
Debt-to-assets ratio increased	− 0.404*** (0.121)	− 0.192 (0.136)
MP_*t* − 2_ × debt-to-assets ratio increased	0.0350*** (0.0102)	0.0200* (0.0114)
Observations	7425	6677
Country × sector FE	Yes	Yes
Time FE	Yes	Yes
Bank controls	Yes	Yes
Macro controls	Yes	Yes
Other firm controls	Yes	Yes

The dependent variable in column 1 is the probability of being discouraged only, and that in column 2 is the probability of being credit constrained (excluding discouraged). The variable MP_*t* − 2_ is the 1-year lag (equivalent to two 6-month survey waves). Debt-to-assets ratio increased is a categorical variable which is equal to 1 if the firm’s debt-to-assets ratio increased, and 0 if it remained the same or decreased in the previous 6 months. Firm-level controls include dummies for firm size, age, industry, turnover and ownership. Bank controls (non-performing loans and tier 1 capital ratio) and macro controls (inflation and unemployment) are lagged by 1 year (equivalent to two survey waves). Robust standard errors are in the parentheses.

*Significance at 10%

***Significance at 1%

**Table 9:** H2 robustness by the type of credit constraint

Credit-constrained excluding discouraged variables	1	2	3	4	5
MP_*t* − 2_	0.0162 (0.0320)	0.0172 (0.0304)	0.0112 (0.0311)	0.000154 (0.0308)	0.00363 (0.0318)
Profit decreased	0.274** (0.127)				
MP_*t* − 2_ × profit decreased	− 0.0152 (0.0106)				
Credit history deteriorated		0.160 (0.219)			
MP_*t* − 2_ × credit history		0.00245 (0.0181)			
Own outlook deteriorated			0.268* (0.140)		
MP_*t* − 2_ × own outlook			− 0.00723 (0.0117)		
Own capital deteriorated				− 0.139 (0.193)	
MP_*t* − 2_ × own capital				0.0249 (0.0162)	
Innovation					0.00305 (0.130)
MP_*t* − 2_ × innovation					0.00356 (0.0108)
Observations	6712	6753	6711	6731	6789
Country × sector FE	Yes	Yes	Yes	Yes	Yes
Time FE	Yes	Yes	Yes	Yes	Yes
Bank controls	Yes	Yes	Yes	Yes	Yes
Macro controls	Yes	Yes	Yes	Yes	Yes
Other firm controls	Yes	Yes	Yes	Yes	Yes

The dependent variable in columns 1–5 is the probability of being credit constrained, excluding discouraged borrowers. MP_*t* − 2_ is the 1-year lag (equivalent to two survey waves). Profit decreased, credit history deteriorated, own outlook deteriorated, own capital deteriorated and innovation are all categorical variables which proxy firm risk from the firm’s viewpoint. Firm-level controls include dummies for firm size, age, industry, turnover and ownership. Bank controls (non-performing loans and tier 1 capital ratio) and macro controls (inflation and unemployment) are lagged by 1 year (equivalent to two survey waves). Robust standard errors are in parentheses.

*Significance at 10%

**Significance at 5%

**Table 10:** H1 robustness by the type of central bank asset

Credit-constrained variables	1	2	3	4	5	6
MP_*t* − 2_ (government securities)	0.0171 (0.0245)	0.0144 (0.0258)	0.00855 (0.0243)			
MP_*t* − 2_ (government securities + MFI securities + MFI lending)				0.0227 (0.0304)	0.0219 (0.0386)	0.00482 (0.0368)
Debt-to-assets ratio increased	− 0.516*** (0.113)	− 0.515*** (0.113)	− 0.416*** (0.107)	− 0.829*** (0.147)	− 0.829*** (0.147)	− 0.694*** (0.141)
MP_*t* − 2_ (government securities) × debt-to-assets ratio increased	0.0479*** (0.00973)	0.0478*** (0.00973)	0.0390*** (0.00925)			
MP_*t* − 2_ (government securities + MFI securities + MFI lending) × debt-to-assets ratio				0.0700*** (0.0119)	0.0700*** (0.0119)	0.0589*** (0.0114)
Observations	8777	8777	8668	8777	8777	8668
Country × sector FE	Yes	Yes	Yes	Yes	Yes	Yes
Time FE	Yes	Yes	Yes	Yes	Yes	Yes
Bank controls	Yes	Yes	Yes	Yes	Yes	Yes
Macro controls	No	Yes	Yes	No	Yes	Yes
Other firm controls	No	No	Yes	No	No	Yes

The dependent variable in columns 1–6 is the probability of being credit constrained. Monetary policy variable in columns 1–3 is the 1-year lag (equivalent to two survey waves) of the logarithm of holdings of government debt securities from individual central bank balance sheet for stressed countries, while in columns 4–6 is the 1-year lag (equivalent to two survey waves) of the logarithm of the sum of holdings of government debt securities, debt securities issued by MFI and loans to MFI from individual central bank balance sheet for stressed countries. Debt-to-assets ratio increased is a categorical variable which is equal to 1 if the firm’s debt-to-assets ratio increased, and 0 if it remained the same or decreased in the previous 6 months. Firm-level controls include dummies for firm size, age, industry, turnover and ownership. Bank controls (non-performing loans and tier 1 capital ratio) and macro controls (inflation and unemployment) are lagged by 1 year (equivalent to two survey waves). Robust standard errors are in the parentheses.

***Significance at 1%

**Table 11:** H2 robustness by the type of central bank asset (MP = government securities)

Credit-constrained variables	1	2	3	4	5	6	7	8	9	10
MP_*t* − 2_ (government securities)	0.0177 (0.025)	0.00980 (0.024)	0.0124 (0.025)	0.00619 (0.023)	− 0.0422* (0.025)	− 0.0413* (0.023)	0.00971 (0.025)	0.0056 (0.024)	0.0290 (0.026)	0.0181 (0.024)
Profit decreased	0.125 (0.105)	0.100 (0.100)								
MP_*t* − 2_ × profit decreased	0.0038 (0.009)	0.0023 (0.009)								
Credit history deteriorated			− 0.158 (0.159)	− 0.160 (0.154)						
MP_*t* − 2_ × credit history			0.038*** (0.0136)	0.034** (0.0132)						
Own outlook deteriorated					0.0568 (0.108)	− 0.004 (0.105)				
MP_*t* − 2_ × own outlook					0.017* (0.009)	0.019** (0.009)				
Own capital deteriorated							0.0829 (0.150)	0.001 (0.144)		
MP_*t* − 2_ × own capital							0.017 (0.013)	0.019 (0.013)		
Innovation									− 0.127 (0.113)	− 0.157 (0.107)
MP_*t* − 2_ × innovation									0.012 (0.009)	0.015 (0.009)
Observations	8836	8726	8896	8779	8826	8707	8849	8734	8943	8820
Country × sector FE	Yes	Yes	Yes	Yes	Yes	Yes	Yes	Yes	Yes	Yes
Time FE	Yes	Yes	Yes	Yes	Yes	Yes	Yes	Yes	Yes	Yes
Bank controls	Yes	Yes	Yes	Yes	Yes	Yes	Yes	Yes	Yes	Yes
Macro controls	Yes	Yes	Yes	Yes	Yes	Yes	Yes	Yes	Yes	Yes
Firm controls	No	Yes	No	Yes	No	Yes	No	Yes	No	Yes

The dependent variable in columns 1–10 is the probability of being credit constrained. Monetary policy variable in columns 1–10 is the 1-year lag (equivalent to two survey waves) of the logarithm of holdings of government debt securities from individual central bank balance sheet for stressed countries. Profit decreased, credit history deteriorated, own outlook deteriorated, own capital deteriorated and innovation are all categorical variables which proxy firm risk from the firm’s viewpoint. Firm-level controls include dummies for firm size, age, industry, turnover and ownership. Bank controls (non-performing loans and tier 1 capital ratio) and macro controls (inflation and unemployment) are lagged by 1 year (equivalent to two survey waves). Robust standard errors are in parentheses.

*Significance at 10%

**Significance at 5%

***Significance at 1%

**Table 12:** H2 robustness by the type of central bank asset (MP = government securities + MFI securities + MFI lending)

Credit-constrained variables	1	2	3	4	5	6	7	8	9	10
MP_*t* − 2_ (government + MFI securities + MFI lending)	0.0383 (0.0378)	0.0197 (0.0364)	0.0302 (0.0368)	0.0134 (0.0354)	− 0.0387 (0.0369)	− 0.0467 (0.0355)	0.0212 (0.0373)	0.00757 (0.0360)	0.0479 (0.0381)	0.0246 (0.0364)
Profit decreased	0.0518 (0.137)	0.0777 (0.132)								
MP_*t* − 2_ × profit decreased	0.00945 (0.0111)	0.00403 (0.0107)								
Credit history deteriorated			− 0.198 (0.212)	− 0.144 (0.205)						
MP_*t* − 2_ × credit history			0.0387** (0.0169)	0.0303* (0.0164)						
Own outlook deteriorated					0.0818 (0.144)	0.0221 (0.139)				
MP_*t* − 2_ × own outlook					0.0138 (0.0116)	0.0155 (0.0113)				
Own capital deteriorated							− 0.0490 (0.209)	− 0.101 (0.201)		
MP_*t* − 2_ × own capital							0.0266 (0.0169)	0.0255 (0.0163)		
Innovation									− 0.177 (0.144)	− 0.189 (0.138)
MP_*t* − 2_ × innovation									0.0154 (0.0116)	0.0167 (0.0111)
Observations	8836	8726	8896	8779	8826	8707	8849	8734	8943	8820
Country × sector FE	Yes	Yes	Yes	Yes	Yes	Yes	Yes	Yes	Yes	Yes
Time FE	Yes	Yes	Yes	Yes	Yes	Yes	Yes	Yes	Yes	Yes
Bank controls	Yes	Yes	Yes	Yes	Yes	Yes	Yes	Yes	Yes	Yes
Macro controls	Yes	Yes	Yes	Yes	Yes	Yes	Yes	Yes	Yes	Yes
Other firm controls	No	Yes	No	Yes	No	Yes	No	Yes	No	Yes

The dependent variable in columns 1–10 is the probability of being credit constrained. Monetary policy variable is the 1-year lag (equivalent to two survey waves) of the logarithm of the sum of holdings of government debt securities, debt securities issued by MFI and loans to MFI from individual central bank balance sheet for stressed countries. Profit decreased, credit history deteriorated, own outlook deteriorated and own capital deteriorated are all categorical variables which proxy firm risk from the firm’s viewpoint. Innovation is a categorical variable, which proxies if the firm innovated in the previous 6 months, and is a measure of firm risk. Firm-level controls include dummies for firm size, age, industry, turnover and ownership. Bank controls (non-performing loans and tier 1 capital ratio) and macro controls (inflation and unemployment) are lagged by 1 year (equivalent to two survey waves). Robust standard errors are in parentheses.

*Significance at 10%

**Significance at 5%

## Footnotes

1 Following Ferrando et al. (2017), Acharya et al. (2018) and Kaya and Masetti (2018), we define the countries in the periphery as stressed countries and include Greece, Ireland, Italy, Portugal and Spain, i.e. those countries with the highest average sovereign yields during the 2009–2012 period.

2 It should be noted that the ECB had initiated UMP with the securities market programme (SMP) from 2010 and outright monetary transactions (OMTs) from 2012. Both programmes were aimed at intervening in stressed bond markets whereby the ECB bought or signalled that they would buy risky sovereign debt denominated in stressed countries to safeguard the appropriate monetary policy transmission and the singleness of the monetary policy across the euro area.

3 For a comprehensive analysis of negative interest rates, see Boucinha et al. (2020) and Altavilla et al. (2022).

4 For a comprehensive assessment of TLTROs, see Afonso and Sousa-Leite (2020) and Andreeva and Garcia-Posada (2021).

5 See ECB (2015) on the role of central bank balance sheets as a monetary policy tool (https://www.ecb.europa.eu/pub/pdf/other/art01_eb201504.en.pdf).

6 Similar models that aim to capture reactions of financially constrained firms to monetary policy are highlighted in Bernanke and Gertler (1989) and Bernanke et al. (1999) that are consistent with the financial frictions literature. The theories on financial frictions suggest the determinants of firm financing constraints are determined by firm characteristics such as age, size and ownership structure.

7 SAFE’s main results are published every 6 months. For more information on the survey, see https://www.ecb.europa.eu/stats/ecb surveys/safe/html/index.en.html.

8 Given this, we conduct analysis in robustness checks excluding discouraged borrowers.

9 To examine the effect of sectors on credit constraints, we use three major activities applied in the SAFE: industry, construction and trade. Enterprises from mining and quarrying; manufacturing; and electricity, gas, steam, and air conditioning supply; and water supply, sewerage, waste management and remediation activities were combined under “Industry”. “Construction” is simply construction. “Trade” includes wholesale and retail trade, repair of motor vehicles, motorcycles and personal and household goods. The statistical stratification was based on economic activities at the one-digit level of the European Nomenclature of Economic Activities (NACE) classification, according to Rev. 2, and excludes businesses operating in agriculture, public administration and financial services (ECB, 2022b). The NACE classification also includes other services; however, there are no observations for other services in our sample, and hence, we exclude this.

10 Size is measured by both employee numbers and turnover as defined by the European Commission (2003). Micro firms are defined as having turnover less than or equal to 2 million, small firms as having turnover greater than 2 and less than or equal to 10 million, medium firms as having turnover greater than 10 and less than or equal to 50 million and large firms as having turnover greater than 50 million. Since our analysis focuses on SMEs, we exclude large firms.

11 Firms are also categorised by size based on turnover. Figures 6 and 7 in Appendix 3 show the coefficient plots for credit constrained for H1 and H2.

12 We consider firms under 5 years as our sample and include only 0.8% of firms that are less than 2 years old.

13 Betz and De Santis (2022) show that the corporate sector purchase programme (CSPP) led to larger companies moving towards issuance of debt securities away from bank loans and banks substituted towards lending to smaller companies.

14 For example, finance sources for micro firms in our sample were credit lines (30.7%), bank loans (27.8%), trade credit (18.3%), retained earnings (6.4%), debt securities (2.9%) and equity (0.3%).

15 See the communication from the European Commission on the “Temporary Framework for State aid measures to support the economy in the current COVID-19 outbreak” at https://eur-lex.europa.eu/legal-content/EN/TXT/HTML/?uri=CELEX:52020XC0320(03)&from=EN. For a broader view, see OECD (2021)  which offers a cross-country analysis of SME policy responses during the survival phase.
